# Mapping two decades of research on climate change and women’s reproductive health: A bibliometric analysis (2000–2024)

**DOI:** 10.1177/17455057261442096

**Published:** 2026-04-17

**Authors:** Mudassar Aziz, Gulnaz Anjum, Abdul Rehman Nawaz

**Affiliations:** 1 University of Oslo, Norway; 2 Central European University Private University – CEU GmbH, Vienna, Austria

**Keywords:** climate change, women’s health, maternal health, preterm birth, bibliometric analysis, climate justice

## Abstract

**Background:**

Climate change is increasingly recognized as a determinant of reproductive and maternal health, yet the scope, evolution, and structural dynamics of this research domain remain underexplored. Bibliometric approaches can clarify thematic priorities, geographic imbalances, and knowledge gaps.

**Objectives:**

This study systematically maps the global research landscape on climate change and women’s reproductive and maternal health from 2000 to 2024, examining temporal trends, thematic clusters, citation networks, collaboration patterns, and geographic distributions, with a particular focus on epistemic and equity-related imbalances.

**Design:**

Bibliometric analysis of 378 peer-reviewed publications indexed in Scopus and Web of Science, combining performance analysis with science mapping techniques.

**Methods:**

Datasets were harmonized and analyzed using Biblioshiny, VOSviewer, and pyBibX. Analyses included co-occurrence mapping, trigram analysis, keyword evolution, bibliographic coupling, and citation and collaboration network analysis. Through co-occurrence mapping and trigram analysis, we identified dominant biomedical framings while revealing marginalized perspectives; through collaboration and citation network analysis, we traced how knowledge production is concentrated in the Global North despite vulnerabilities being greatest in the Global South.

**Results:**

The field has expanded rapidly since 2020, consolidating around biomedical endpoints such as preterm birth, fertility, and maternal morbidity. Research output and influence are dominated by high-income countries, particularly the United States, United Kingdom, China, and Australia. Female scholars (e.g., Kovats, Bonell, Filippi) are central to collaboration networks, but Southern contributions remain underrepresented and often embedded in asymmetrical North–South partnerships. Journal productivity is led by *International Journal of Environmental Research and Public Health* and *Environmental Research*. The field remains predominantly biomedical, with limited integration of justice-oriented and intersectional frameworks.

**Conclusions:**

Research on climate change and women’s health has matured substantially, establishing causal pathways between environmental stressors and adverse reproductive outcomes. However, epistemic inequities persist, with knowledge production concentrated in the Global North and underrepresentation of Global South leadership. Advancing the field requires equity-driven collaborations, interdisciplinary approaches, and the integration of feminist and decolonial perspectives to ensure inclusive and just knowledge production.

## Plain language summary

**Climate change and women’s reproductive health: What we know after 20** **years of research**

Climate change is often discussed in terms of rising temperatures, extreme weather, and environmental damage. Less attention is given to how it affects women’s reproductive and maternal health. This study looks at two decades of research (2000–2024) on the topic to understand what scientists know and where gaps remain. We reviewed 378 scientific articles published worldwide. The research shows clear links between climate change and risks such as preterm birth, low birth weight, pregnancy complications, and reduced fertility. These health impacts are most often connected to high heat and air pollution. The number of studies has grown rapidly since 2020, but most are led by researchers in high-income countries, even though the greatest risks are faced by women in low- and middle-income regions. This imbalance limits how well the science reflects the real challenges faced by the most vulnerable populations. Our findings highlight two key needs: more inclusive global research partnerships and a broader perspective that goes beyond medical risks to also consider social inequality and justice. Addressing these gaps will make research more relevant and ensure better protection for women and infants in a changing climate.

## Introduction

Climate change is widely recognized as the greatest public health threat of the 21st century, with profound and multifaceted implications for human health and well-being.^1^ Rising global temperatures, increasing frequency of heatwaves, and deteriorating air quality are directly linked to adverse health outcomes, while indirect effects emerge through food insecurity, displacement, and the erosion of health systems.^1^ Among the populations most affected, women, particularly during pregnancy and reproductive years, face unique and disproportionate risks.^2^ These arise not only from physiological vulnerabilities but also from deeply entrenched social, economic, and structural inequalities.^3–5^

Evidence from epidemiological studies shows consistent associations between maternal exposure to extreme heat or air pollution and adverse outcomes such as preterm birth, low birth weight, stillbirth, and hypertensive disorders.^6–9^ Systematic reviews and meta-analyses underscore the robustness of these findings across contexts, suggesting that reproductive health is a key pathway through which climate change exerts intergenerational impacts.^4,10–12^ At the same time, qualitative research highlights the lived realities of pregnant women in climate-vulnerable regions, where heat exposure exacerbates exhaustion, food insecurity, and limited access to care.^4,13^ This dual lens of biomedical outcomes and social experience reveals that climate change acts as both a physiological stressor and a socio-structural amplifier of existing inequities.

The gendered nature of climate–health interactions is increasingly recognized in global health discourse. Feminist political ecology (FPE) perspectives argue that women’s disproportionate climate risks stem from the intersection of biology, labor roles, and systemic marginalization, particularly in low- and middle-income countries.^12,14^ Yet, despite growing concern, research on climate change and women’s health remains fragmented. Much of the literature is concentrated in high-income countries, with limited leadership from the Global South, where climate impacts are most acute.^15,16^ This imbalance reflects broader epistemic inequalities in global health research, where Northern institutions often set the agenda while Southern contexts are positioned primarily as sites of data collection.^17,18^

Against this backdrop, bibliometric analysis provides a powerful tool to systematically map the knowledge domain, identify thematic clusters, and highlight gaps in evidence and representation.^19^ While individual studies and reviews have examined specific health outcomes such as preterm birth or stillbirth, few have taken a comprehensive view of how climate change and women’s health intersect as an emergent research field. Bibliometric approaches enable an overview of publication trends, citation networks, institutional leadership, and geographic distribution, thereby offering insights not only into what is known but also into who produces this knowledge and under what structural conditions.^20,21^

This study therefore undertakes the first large-scale bibliometric analysis of research on climate change and women’s health from 2000 to 2024. By integrating data from both Web of Science (WoS) and Scopus, we provide a consolidated mapping of the field’s evolution, major thematic areas, and global patterns of collaboration. The study seeks to answer three core questions: (1) How has scientific production on climate change and women’s health evolved over time? (2) What are the dominant themes, outcomes, and methodological approaches? and (3) How are authorship, institutional contributions, and geographic representation distributed across the field? Addressing these questions contributes not only to advancing the evidence base on climate and health but also to illuminating epistemic inequities that must be confronted in pursuit of climate justice and reproductive health equity.

## Rationale and literature review

The health impacts of climate change are increasingly recognized across epidemiology, medicine, and public health. Global assessments emphasize that climate-related exposures—particularly extreme heat, air pollution, vector-borne disease expansion, and disruptions to food and water systems—are already undermining health outcomes worldwide.^1^ Climate-sensitive health burdens are not evenly distributed; rather, they cluster disproportionately in regions with limited adaptive capacity, weak health infrastructure, and high preexisting vulnerability.^22,23^ The Lancet Countdown reports consistently demonstrate that without rapid mitigation and adaptation, climate change will reverse decades of progress in global health.^1^

Among health domains, maternal and reproductive health has emerged as a critical site of climate impact. A growing body of epidemiological evidence links maternal exposure to heat and air pollution with adverse birth outcomes such as preterm birth, low birth weight, and stillbirth.^4,6–8^ Systematic reviews and meta-analyses provide consistent evidence across multiple contexts, with some studies estimating increased risks of preterm birth of 10%–20% during periods of high temperature exposure.^9–11^ Other studies show synergistic effects of climate stressors—for example, combined heat and air pollution exposures significantly elevate maternal cardiovascular stress and pregnancy complications.^4,8^ These findings underscore reproductive health as a key pathway through which climate impacts are transmitted across generations.

While biomedical studies establish causal pathways between climate exposures and health outcomes, FPE and global health scholarship highlight that women’s vulnerabilities are shaped by intersecting social and structural determinants.^14,24^ Gendered divisions of labor, unequal access to health services, and structural constraints on mobility and adaptation exacerbate climate-related health risks.^3,25^ In many low- and middle-income contexts, women face disproportionate burdens of unpaid care work, limited autonomy in health decision-making, and higher rates of occupational exposure in agriculture and informal sectors.^4,16^ These dynamics not only amplify direct physiological risks but also deepen indirect effects such as food insecurity, malnutrition, and psychosocial stress.^13^ Intersectional analyses further reveal that age, poverty, disability, and displacement compound gendered health vulnerabilities, pointing to the need for integrated and justice-oriented approaches.^15,26^

Despite rising attention to climate–health linkages, the literature on women’s health within this context remains uneven and fragmented. Bibliometric evidence shows that research outputs are heavily concentrated in high-income countries, even though empirical data often derive from climate-vulnerable regions in the Global South.^16,27^ Such patterns reflect broader epistemic inequalities, whereby Global North institutions dominate authorship, funding, and agenda-setting, while Global South contributions remain underrepresented.^14,18^ As a result, locally grounded perspectives on women’s health and climate adaptation are marginalized, and global evidence bases risk reproducing colonial hierarchies of knowledge.

While several systematic reviews have examined specific health outcomes, such as preterm birth, stillbirth, or fertility decline, few have mapped the field as an integrated domain that links climate change with women’s health in all its complexity. Bibliometric methods provide a means to systematically assess the intellectual and structural landscape of research, including publication growth, thematic evolution, collaboration patterns, and citation networks.^21,28,29^ By synthesizing data from WoS and Scopus, this study not only captures the breadth of evidence but also reveals the epistemic geographies of knowledge production. Such insights are essential for identifying gaps, amplifying marginalized perspectives, and advancing a more inclusive, justice-oriented research agenda on climate change and women’s health. While Zhang et al.^30^ recently published a bibliometric analysis examining climate change impacts on pregnancy and neonatal outcomes, our study makes three complementary contributions that distinguish it from existing work. First, we employed more targeted and precise search filters specifically designed to capture women’s reproductive health literature, ensuring comprehensive coverage of reproductive system function, maternal health, and climate impacts across the reproductive lifecourse. Second, we are the first to analyze this field through explicit theoretical frameworks of epistemic justice, FPE, and decolonial knowledge production, operationalizing these lenses to examine how bibliometric patterns manifest structural power inequities and moving beyond descriptive mapping to critical structural analysis. Third, we employ methodological innovations including database merging (WoS + Scopus), multi-tool triangulation (pyBibX, Biblioshiny, VOSviewer), and granular authorship hierarchy analysis that reveals how North–South collaborations reproduce colonial dynamics through corresponding author concentration and middle-author relegation of Southern scholars. In summary, while Zhang et al.^30^ provide valuable descriptive mapping of climate-pregnancy research, our study uniquely analyzes why knowledge production is structured as it is through refined search methodology, critical theoretical grounding, and equity-focused analysis.

## Theoretical framework

This study is grounded in three interconnected theoretical frameworks that inform our interpretation of bibliometric patterns: epistemic justice, FPE, and decolonial knowledge production.

Epistemic justice, as articulated by Fricker^31^ and extended by de Sousa Santos^18^ through the concept of “epistemologies of the South,” refers to fairness in the production, validation, and circulation of knowledge. Epistemic injustice manifests in two primary forms: testimonial injustice (when certain voices are systematically discredited) and hermeneutical injustice (when marginalized groups lack the conceptual resources to articulate their experiences). In global health research, epistemic injustice is structural: knowledge produced in the Global North is positioned as universal and authoritative, while Southern scholarship is often relegated to the status of “local knowledge” or case material.^16^

In this study, we operationalize epistemic injustice through examination of: (a) authorship hierarchies, who occupies first, last, and corresponding author positions; (b) institutional affiliations, which universities and research centers dominate knowledge production; (c) citation patterns, whose work is recognized as foundational and canonical; and (d) collaboration asymmetries, whether North–South partnerships reflect equitable coproduction or extractive relationships where Southern partners contribute data while Northern institutions control interpretation and publication.

FPE provides a critical lens for understanding how environmental burdens and climate vulnerabilities are gendered and intersectional.^14,24^ FPE argues that women’s disproportionate climate risks cannot be understood through biological vulnerability alone; rather, they emerge from intersecting systems of patriarchy, capitalism, and colonialism that structure women’s labor, mobility, autonomy, and access to resources. FPE emphasizes that vulnerability is produced, not natural, it is the outcome of structural inequalities in power, resources, and decision-making.

We operationalize FPE in this bibliometric analysis by examining: (a) whether research themes prioritize biomedical outcomes (e.g., preterm birth) over socio-structural determinants (e.g., gendered labor, poverty, migration); (b) whether keywords and abstracts reflect intersectional frameworks that account for how gender intersects with race, class, and geography; and (c) whether the field engages with feminist scholarship or remains confined to biomedical epidemiology.

Decolonial theory challenges the ongoing coloniality of knowledge, the ways in which colonial power relations persist in contemporary academic structures.^18,32^ In global health research, coloniality manifests through the following: Northern institutions setting research agendas based on their priorities; Southern contexts being framed as sites of risk and data extraction rather than as sources of theory and leadership; and Southern scholars being included primarily in subordinate authorship positions or excluded entirely.^33^

In this study, we operationalize decolonial critique through the analysis of: (a) geographic distribution of research leadership, which countries lead publications and where are corresponding authors located; (b) research agenda–setting, whether research priorities reflect Northern concerns (e.g., quantifiable biomedical outcomes) or Southern priorities (e.g., structural violence, climate justice, community resilience); and (c) collaboration patterns, whether they reflect genuine partnership or reproduce colonial hierarchies.

These three frameworks converge in revealing how bibliometric patterns are not merely descriptive statistics but material manifestations of power. High citation counts for Northern scholars reflect epistemic authority; biomedical dominance in keywords reflects disciplinary hierarchies that privilege quantifiable endpoints over qualitative and structural analyses; and collaboration networks centered in the Global North reflect resource asymmetries and historical legacies of colonialism. By applying these frameworks, we transform bibliometric data into evidence of epistemic inequality.

## Methodology

### Eligibility criteria

The inclusion criteria for this bibliometric analysis were as follows: peer-reviewed articles and reviews published between January 1, 2000, and December 31, 2024; documents containing climate change and women’s health-related terms in the title, abstract, or author keywords; and full-text availability in English. Exclusion criteria included conference proceedings, editorials, letters, book chapters, dissertations, and non-peer-reviewed materials. Records that were not aligned with the thematic scope excluded after title and abstract screening. The removal of duplicates across databases occurred at the last stage prior to analysis with Python library.

### Information sources

For the current study, we employed two major multidisciplinary databases. WoS Core Collection and Scopus were selected due to their comprehensive coverage and established reliability for bibliometric research.^34^ Researchers have used Scopus^35^ and WoS^36^ independently but recently they started merging these two database as it leads to much richer results.^29^ Keeping in line with recent research trends, our search was executed on August 2, 2025, at 9:46 pm. Both databases were queried independently in the same period, and the exported files were later harmonized and merged to construct the final dataset.

### Search strategy

We developed advanced search queries to capture the intersection of climate change and women’s health. The Scopus query targeted the title, abstract, and keyword fields, while the WoS query used the topic search (TS) function. Both included terms for climate change and variability (“climate change,” “global warming,” “climate crisis,” “heat stress,” “climate variability,” “environmental change”), reproductive and maternal health (“reproductive health,” “maternal health,” “pregnancy,” “fertility,” “miscarriage,” “birth outcomes,” “menstruation,” “family planning”), and gender (“women,” “female,” “girls,” “gender”).

The queries we put are given below:

**WoS**
TS=((“climate change” OR “global warming” OR “climate crisis” OR “heat stress” OR “climate variability” OR “environmental change”)
AND (“reproductive health” OR “maternal health” OR “pregnancy” OR “fertility” OR “miscarriage” OR “birth outcomes” OR “menstruation” OR “family planning”)
AND (women OR female OR girls OR gender))
**Scopus**
TITLE-ABS-KEY((“climate change” OR “global warming” OR “climate crisis” OR “heat stress” OR “climate variability” OR “environmental change”)
AND (“reproductive health” OR “maternal health” OR “pregnancy” OR “fertility” OR “miscarriage” OR “birth outcomes” OR “menstruation” OR “family planning”)
AND (women OR female OR girls OR gender))

### Selection process

The initial WoS search yielded 892 records, and Scopus returned 2489. Filters were applied to restrict document type to articles and reviews, language to English, and in WoS, research categories were further refined to “Environmental Sciences,” “Public Environmental Occupational Health,” “Environmental Studies,” “Multidisciplinary Sciences,” “Demography,” “Economics,” “Social Issues,” “Family Studies,” “Sociology,” “Political Science,” “Health Policy Services,” “Women Studies,” “Social Sciences Interdisciplinary,” and for Scopus it was refined to “Medicine,” “Environmental Science,” “Multidisciplinary,” “Social Sciences,” “Earth and Planetary Sciences,” “Psychology,” “Economics,” “Econometrics and Finance,” “Health Professions.” This filtering reduced the dataset to 294 documents in WoS and 1163 in Scopus. To further enrich the Scopus corpus, it was filtered by keywords and following keywords were removed “Animals,” “Nonhumans,” “Animal,” “Animal Experiment,” “Cattle, Animal Tissue,” “Gene Expression,” “Animal Model,” “Animal Cell,” “Unclassified drug,” “Mouse,” “Blood,” “Mice,” “Chemistry.” Effective dataset filtering is essential in bibliometric studies to ensure thematic precision, reduce noise, and enhance the reliability of analytical outcomes.^19^ In the end, a detailed title and abstract screening was done, and 165 records were retained after using Preferred Reporting Items for Systematic Reviews and Meta-Analyses (PRISMA) method from WoS and 308 from Scopus and download at BibTeX (.bib) formats. This study was conducted and reported in accordance with the PRISMA 2020 guidelines.^37^

**Figure 1.**
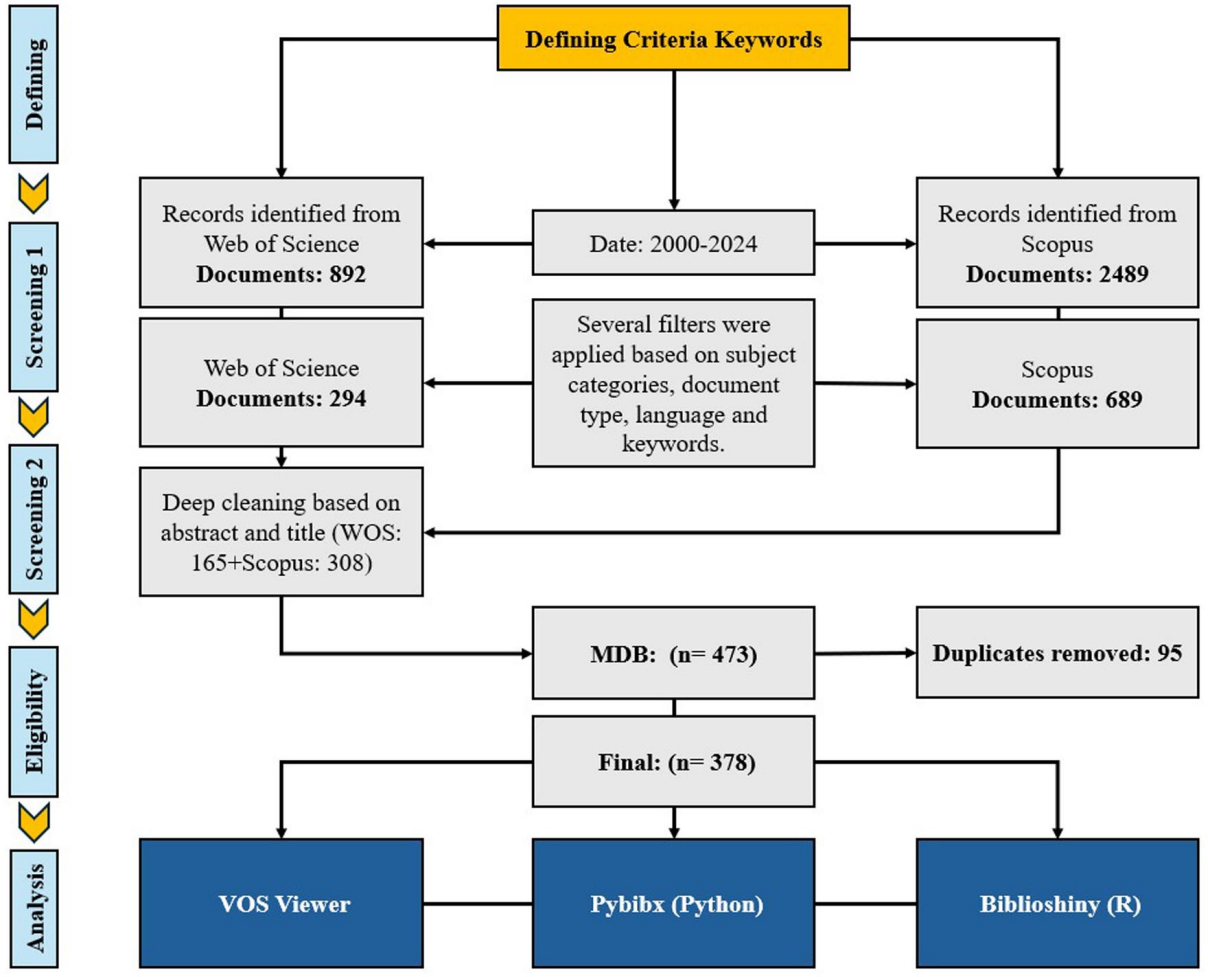
PRISMA diagram for the selection of studies. PRISMA: Preferred Reporting Items for Systematic Reviews and Meta-Analyses.

The completed PRISMA checklist is provided as Supplemental Material and the process of selection of papers is also depicted in Figure 1. Table 1 explains the filtering process more thoroughly. Our keyword exclusion strategy was designed to focus the analysis on human reproductive health in the context of climate change, as our research questions concerned the production of knowledge on women’s health rather than mechanistic biological processes. Animal model studies, while scientifically valuable for understanding physiological mechanisms, represent a fundamentally different research paradigm with different authorship patterns, funding sources, and epistemic communities.

**Table 1.** Inclusion and exclusion criteria for documents selection.

Filters	WoS	Scopus
	Documents remaining after filters	
Initial search with timeframe (2000–2024)	892	2489
English	886	2456
Articles and review articles	870	2194
Filtered by categories and subject area	294	1163
Filtered by keyword	—	689 (for Scopus only)
Cleaning (based on title and abstracts)	165	308
Downloaded	165	308

WoS: Web of Science.

However, we recognize that this exclusion may have removed: (a) translational research bridging animal models and human health; (b) studies examining endocrine disruption and reproductive toxicology relevant to climate-related exposures; and (c) mechanistic research that informs human health understanding. The exclusion of “Gene Expression” and “Chemistry” may have particularly eliminated molecular epidemiology studies that examine biological pathways through which climate exposures affect reproductive outcomes.

### Merging the Scopus and WoS datasets

Merging both databases increases the robustness of bibliometric analyses by compensating for disciplinary and indexing biases (Echchakoui, 2020). The BibTeX from Scopus and WoS were uploaded to the Python library to make a merge database (MDB). The WoS dataset (164 unique records after removing 1 duplicate) comprised 140 articles, 22 reviews, and 2 articles in the press. The Scopus dataset contributed 308 unique records (253 articles and 55 reviews). After integration and deduplication, the MDB comprised 378 unique documents: 309 articles, 67 reviews, 2 articles in press. The preliminary report on the merged dataset is given in Table 2.

**Table 2.** Merged metadata report.

Main information	Results
Timespan	2000–2024
Total number of countries	79
Total number of institutions	834
Total number of sources	207
Total number of references	8728
Total number of languages	1
English (No. of docs)	378
Total number of documents	378
Article	311
Review	67
Average documents per author	1.17
Average documents per institution	2.53
Average documents per source	1.83
Average documents per year	18
Total number of authors	1671
Total number of authors keywords	930
Total number of authors keywords plus	2184
Total single-authored documents	44
Total multiauthored documents	334
Average collaboration index	5.13
Max H-index	7

### Rationale for database selection

First, WoS and Scopus provide structured, standardized bibliographic metadata essential for bibliometric analysis, including author affiliations, citation counts, author keywords, and referenced sources in consistent formats that facilitate computational analysis.^38^ Second, both databases have established reliability for citation analysis and network mapping, with documented quality-control processes for indexing.^39^ Third, merging WoS and Scopus has become a best practice in recent bibliometric research to compensate for disciplinary biases inherent in any single database.^17,29^ Fourth, both databases provide the APIs and export functions necessary for computational bibliometric tools like VOSviewer, Biblioshiny, and pyBibX.

### The pybibx: a brief introduction

The pyBibX is a versatile Python library for bibliometric and scientometric analysis that integrates cutting-edge artificial intelligence (AI) tools such as embedding vectors, topic modeling, text summarization, and natural language processing via models like ChatGPT with traditional analytical approaches.^21^ This Python library is open and free to use for public and available on GitHub.^21^ It supports comprehensive exploratory data analysis (EDA), including graphical plots including *n*-gram, word clouds, Sankey diagrams, and evolution plots, alongside network analyses of citation, collaboration, and similarity structures.^21^ This breadth of functionality sets pyBibX apart from tools like Biblioshiny (the GUI for the R package Bibliometrix), which offers robust visual analytics and workflows in a user-friendly web interface but lacks AI-driven text processing capabilities.^21^ Similarly, VOSviewer surpasses in network visualization and clustering of bibliometric data through co-occurrence, citation, and co-citation maps, but does not provide integrated AI-powered summarization or embedding analyses.^21^

### Integrated workflow for bibliometric analysis using pyBibX, Biblioshiny, and VOSviewer

We used a recently developed state of the art Python library (pyBibX) to merge and analyze bibliographic datasets from both Scopus and WoS. We used Example 5 in (WOS + Scopus) Google Colab web interface. It can be run alternatively on Jupyter Notebook. The pyBibx library is available at the following web address: https://pypi.org/project/pyBibX/. We first aggregated the .bib files exported from Scopus and WoS to make an MDB, which we have already described. Since our data were already cleaned, we proceeded to the metadata profiling (including authors, institutions, sources, and keywords), EDA, and network construction including citation, collaboration, and similarity networks.

In parallel, the bibliographic records exported in a .bib format were imported into Biblioshiny, the R-based web interface of the Bibliometrix package,^40^ to generate Excel files for standardized processing. These Excel files were then merged into a consolidated MDB dataset within Biblioshiny. The merged MDB file was subsequently converted into a .txt format, which served as the input for VOSviewer to conduct the network analyses. This workflow enabled the construction of keyword co-occurrence maps. The combined use of these tools, PyBibX for preprocessing and intelligent handling of metadata, Biblioshiny for structured merging and exporting, and VOSviewer for high-quality visualization,^41^ ensured both the robustness and efficiency of the bibliometric analysis pipeline.

## Results

The annual scientific production on climate change and women’s health remained low until the mid-2010s but has increased substantially in recent years, reaching 96 publications in 2024. Citation activity also showed a similar trend where with reaching peaks in 2010, 2017, and particularly in 2020, when yearly citations surpassed 1700. However, if we look at the country level, the United States has the highest citation share (4543), followed by the United Kingdom, Australia, and China, with notable though comparatively smaller contributions from South Africa and Bangladesh. This distribution shows the concentration of research influence in high-income countries, while also revealing the empirical importance of climate-vulnerable regions in the Global South. See Supplemental Figures S1–S3 for more details.

**Figure 2.**
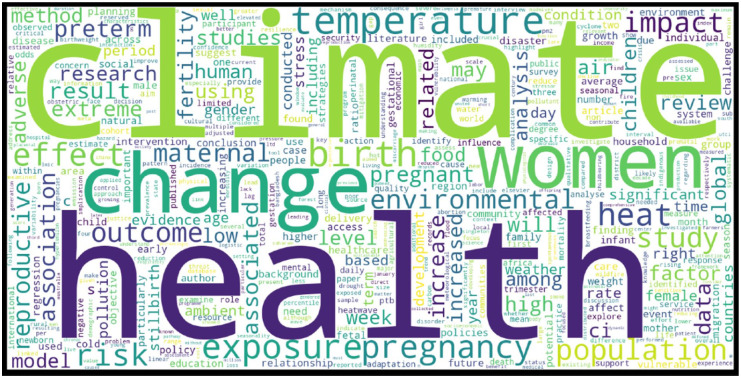
Word Cloud and 3-gram plot based on abstract.

The upper part of Figure 2 shows the most frequent terms appearing in the abstracts in our dataset. Unsurprisingly, the most prominent words such as *climate, health, women, change, pregnancy*, and *exposure* show the central focus of this research domain. Terms such as *temperature, risk, birth, reproductive, maternal, infant*, and *heat* also indicate a strong focus on the biological and health outcomes associated with environmental stressors. Meanwhile, words like *impact, effect, outcome, population*, and *study* point toward a literature that is both impact-oriented and empirically driven. The co-occurrence of *food, fertility, preterm, low birth weight*, and *air pollution* signal that research has expanded beyond general health impacts to include reproductive outcomes and determinants of maternal and child health. Based on word cloud visualization, we can say that the field is structured around three overlapping thematic axes: (i) environmental stressors (climate change, temperature, air pollution, heat waves), (ii) women’s reproductive and maternal health outcomes (pregnancy, birth, fertility, maternal and infant risks), and (iii) population-level consequences (vulnerability, risk, exposure, adaptation). See the complete word cloud in Supplemental Table S1 and Supplemental Figure S4 for the Tree map of author keywords in the Supplemental Material.

**Figure 3.**
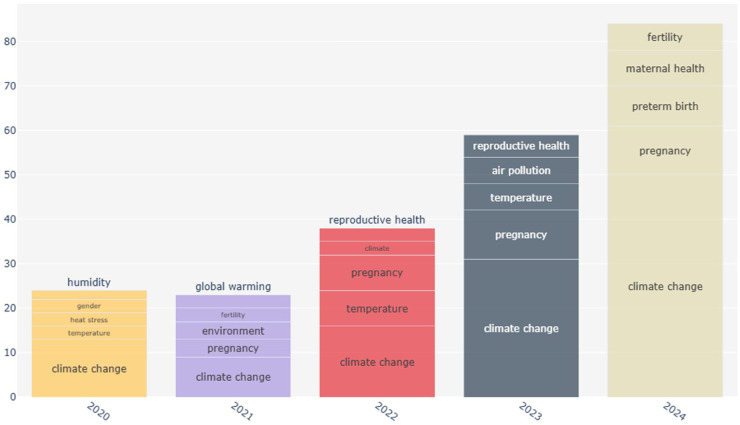
Evolution plot 2020–2024 authors keywords.

Figure 3 shows the temporal evolution of authors’ keywords from 2020 to 2024. The figure clearly shows a shift from broad environmental terms such as humidity, heat stress, temperature, and global warming toward more outcome-specific health themes. While climate change remained a constant anchor across the years, yet the vocabulary gradually expanded to include reproductive health, pregnancy, and air pollution. By 2024, the literature further specialized with keywords such as “fertility,” “maternal health,” and “preterm birth.”

**Figure 4.**
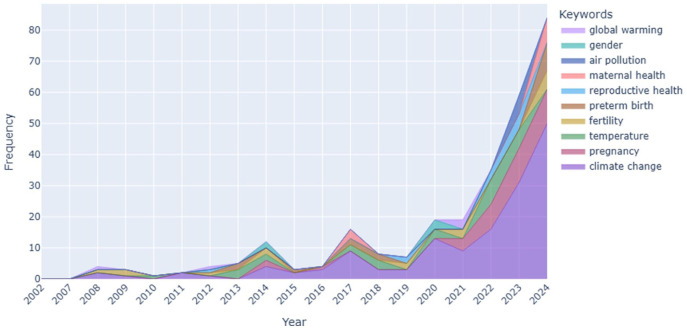
Author keywords evolution.

Figure 4 shows the evolution of author keywords over time. Before 2010, we do not see any peaks and literature production remain limited, but after 2010, we see three major peaks in 2014, 2017, and 2020, respectively. A sharp rise from 2021 onward (COVID-19) is still continue. Climate change remained the dominant term across the period, but keywords such as pregnancy, temperature, reproductive health, and maternal health have gained increasing prominence in recent years. The emergence of terms such as “preterm birth,” “fertility,” and “air pollution” show the field’s consolidation around reproductive and maternal health risks, consistent with epidemiological reviews linking extreme heat, poor air quality, and climate stressors to adverse pregnancy and child health outcomes. Supplemental Figure S5 created with VOSviewer also shows the evolution of author keywords overtime.

**Figure 5.**
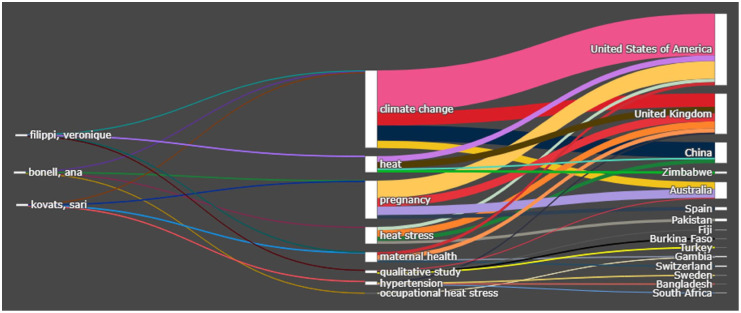
Sankey diagram (author/author keywords/countries).

Figure 5, which is a Sankey diagram, links authors, author keywords, and countries. It shows how scholarly contributions are distributed across themes and geographic contexts. Prominent authors such as Véronique Filippi, Ana Bonell, and Sari Kovats have contributed to multiple themes, particularly around climate change, heat, pregnancy, and heat stress. These themes are strongly connected to research works from high-income countries such as the United States, the United Kingdom, and Australia. This indicates that much of the knowledge production in this domain is concentrated in the Global North. At the same time, studies on maternal health, hypertension, and occupational heat stress were conducted in Zimbabwe, Pakistan, Bangladesh, Burkina Faso, and South Africa. It shows enduring asymmetries in global knowledge production, where the research agenda is largely set by institutions in the Global North, while empirical evidence and case material are disproportionately extracted from climate-vulnerable regions of the Global South. Such dynamics reproduce a colonial division of labor in which Northern scholars retain epistemic authority and resources, whereas Southern contexts are framed primarily as sites of risk and data collection.

**Figure 6.**
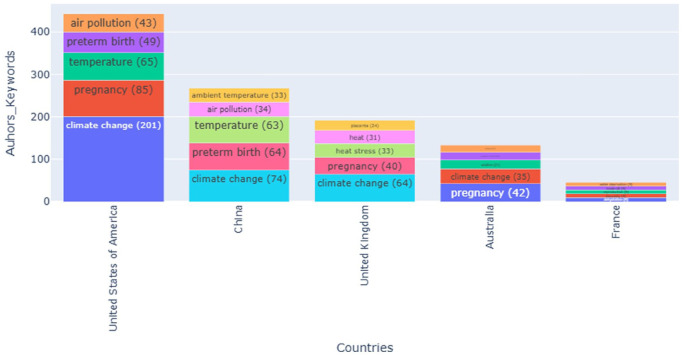
Distribution of author keywords per countries.

Figure 6 shows the distribution of author keywords across leading contributing countries. Research from the United States dominates, with *climate change* (201), *pregnancy* (85), *temperature* (65), *preterm birth* (49), and *air pollution* (43) as recurrent keywords. China also features prominently, where terms such as *temperature* (63), *climate change* (74), and *air pollution* (34) showcase a focus on environmental exposures and reproductive health risks. In the United Kingdom, *climate change* (64), *pregnancy* (40), and *heat stress* (33) emerge as central themes, while Australia is distinguished by its emphasis on *pregnancy* (42) alongside *climate change* (35). France, by contrast, contributes far fewer publications, with dispersed keywords and limited thematic concentration.

**Figure 7.**
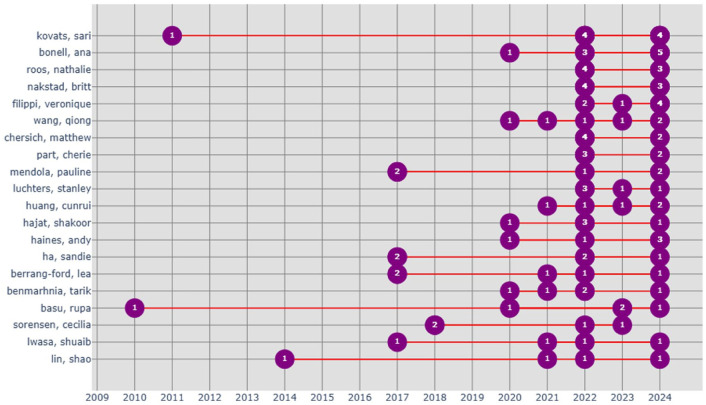
Longitudinal productivity of top 20 authors (2009–2024).

Figure 7 shows the productivity of the top 20 authors over time by showing both entry points into the field and patterns of sustained publication. A few scholars, such as Sari Kovats (associate professor at London School of Hygiene & Tropical Medicine) and Rupa Basu (California Office of Environmental Health Hazard Assessment), appear as early contributors with work dating back to 2009 and 2010, respectively, and their foundational role of environmental epidemiology in linking climate exposures with health outcomes. However, most authors entered the field only after 2016, with notable expansion occurring from 2020 onward. This surge coincides with the broader rise of climate change and health as a global policy and research priority.

**Figure 8.**
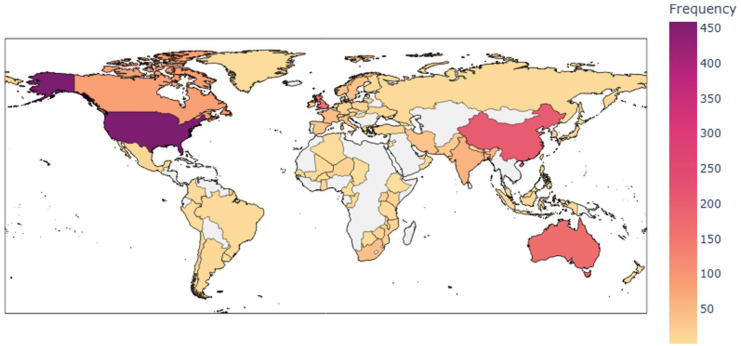
Global distribution of research productivity.

Figure 8 shows the global distribution of research productivity based on the number of documents in MDB. There is a clear concentration of output in high-income countries. The United States, as always, dominates the field, followed by the United Kingdom, China, and Australia, while most of Africa, South America, and parts of Asia remain sparsely represented. This geographic imbalance shows that knowledge production is clustered in well-resourced Northern institutions, even though climate-related health risks disproportionately affect populations in the Global South. The pattern reinforces structural asymmetries in the field, where epistemic authority is consolidated in the North while empirical vulnerabilities in the South remain underrepresented in authorship and institutional leadership.

**Figure 9.**
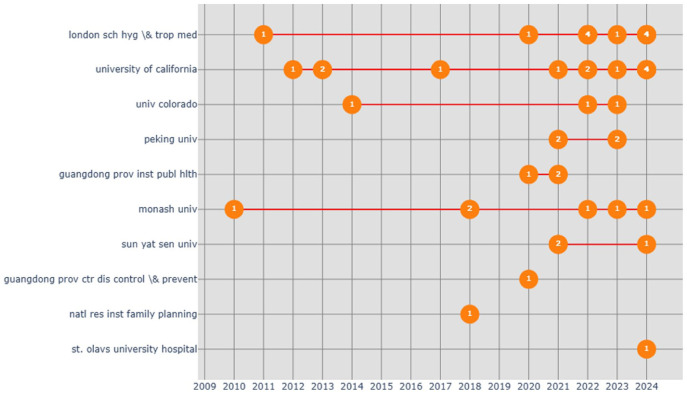
Distribution by institutions productivity over time.

Figure 9 shows the productivity of the top 10 institutions over time, based on documents published for each institution. The London School of Hygiene and Tropical Medicine and the University of California appear as the most consistent contributors, as they maintained their publication activity across multiple years. University of Colorado, Peking University, Monash University, and the Guangdong Provincial Institute of Public Health also show increasing engagement in recent years, which can be interpreted as the global expansion of research capacity in this field. This pattern suggests that a handful of well-established Northern institutions continue to produce knowledge. However, a recent emerging contribution from Chinese and Australian institutions signals a gradual diversification of research leadership beyond traditional centers of expertise.

**Figure 10.**
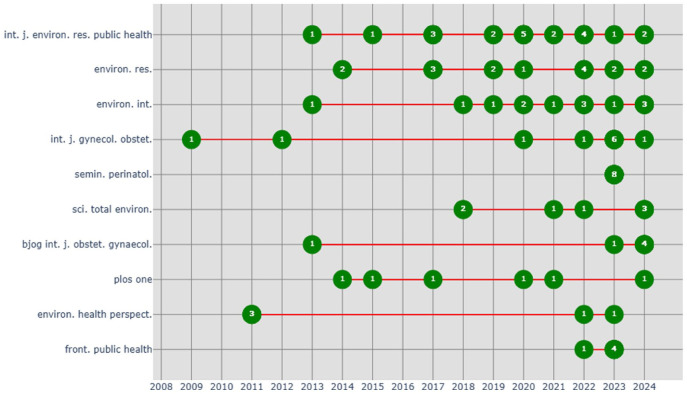
Distribution by sources productivity over time.

Figure 10 shows the productivity of the top 10 most active journals, based on the number of documents published on change between 2008 and 2024. The *International Journal of Environmental Research and Public Health* and *Environmental Research* emerged as leading journals. *Environmental International*, *BJOG: An International Journal of Obstetrics and Gynaecology*, and the *International Journal of Gynecology and Obstetrics* also emerge as important journals. However, more recent contributions from *Frontiers in Public Health* and *Seminars in Perinatology* show the diversification of publication venues. This can be due to quick review and publishing timeframe.

Table 3 shows 10 most highly cited documents in the corpus. Chersich et al. (2020), a systematic review and meta-analysis on high temperatures in pregnancy and risks of preterm birth, low birth weight, and stillbirths, is the most cited contribution (21 citations). Strand et al. (2011) and Zhang et al. (2017) also focus on temperature and seasonality, consolidating epidemiological evidence on adverse birth outcomes, being the second and third, respectively. Seminal works of including Basu et al. (2010) and Strand et al. (2012) establish early evidence linking ambient heat exposure to preterm delivery, while more recent studies by Bekkar et al. (2020) and He et al. (2016) expand this evidence base in the United States and China, respectively. Historical perspectives, such as Bruckner et al. (2014) on cold exposure in Sweden, and broader examinations of climate impacts on birth weight (Deschenes et al., 2009), add more depth to the field.

**Table 3.** Most cited references.

No.	Reference	Author (year)	Citations
1	Associations between high temperatures in pregnancy and risk of preterm birth, low birth weight, and stillbirths: systematic review and meta-analysis	Chersich et al. (2020)	21
2	The influence of season and ambient temperature on birth outcomes: a review of the epidemiological literature	Strand (2011)	20
3	Heat exposure and maternal health in the face of climate change	Kuehn and McCormick (2017)	17
4	Temperature exposure during pregnancy and birth outcomes: an updated systematic review of epidemiological evidence	Zhang et al. (2017)	16
5	High ambient temperature and the risk of preterm delivery	Basu et al. (2010)	15
6	Maternal exposure to ambient temperature and the risks of preterm birth and stillbirth in Brisbane, Australia	Strand et al. (2012)	13
7	Association of air pollution and heat exposure with preterm birth, low birth weight, and stillbirth in the US	Bekkar et al. (2020)	12
8	Ambient temperature and the risk of preterm birth in Guangzhou, China (2001–2011)	He et al. (2016)	12
9	Cold ambient temperature in utero and birth outcomes in Uppsala, Sweden, 1915–1929	Bruckner et al. (2014)	11
10	Climate change and birth weight	Deschenes et al. (2009)	10

**Figure 11.**
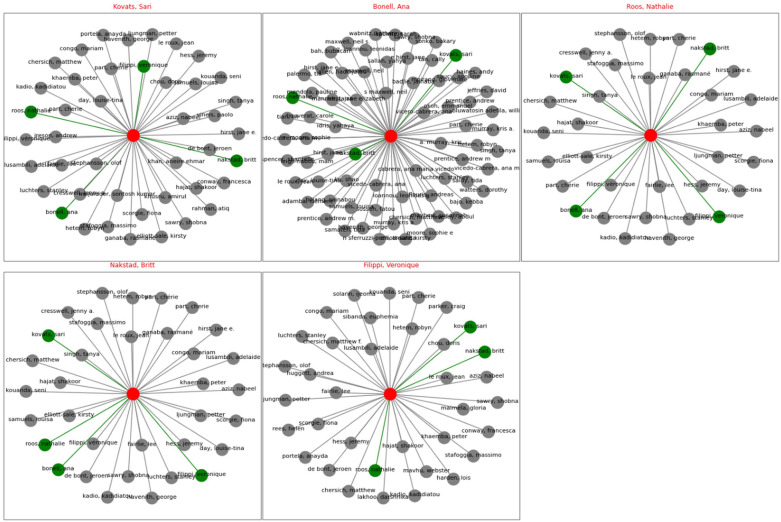
Top 5 author collaboration networks.

The collaboration network plot (Figure 11) shows the coauthorship relationships of the five most prolific authors in the dataset, and it also shows how research communities in this field are structured. Each subplot places one of the top authors. Sari Kovats (associate professor at London School of Hygiene & Tropical Medicine), Ana Bonell (assistant professor at London School of Hygiene & Tropical Medicine), Nathalie Roos (researcher Karolinska Institutet, Sweden), Britt Nakstad (professor at University of Botswana), and Véronique Filippi (professor at London School of Hygiene & Tropical Medicine) are represented at the center (red node), with green nodes marking their direct collaborators and gray nodes representing other authors in the broader network who have not coauthored them in that specific cluster. The visualization shows that while all five scholars maintain extensive collaborations, the density of their networks varies: Bonell’s cluster is particularly wide-ranging, reflecting her engagement in large, multi-institutional projects, while Kovats and Filippi have strong integration into core groups that also include other leading figures such as Chersich and Hajat. By contrast, the networks of Roos and Nakstad, though active, appear less interconnected, pointing to more project-based collaborations. Interestingly, all these five authors are female which shows that female scholars are leading the most exciting research on women reproductive health and climate change. Similar analysis was performed for the top 5 countries. Please see the Supplemental Figure S6.

**Figure 12.**
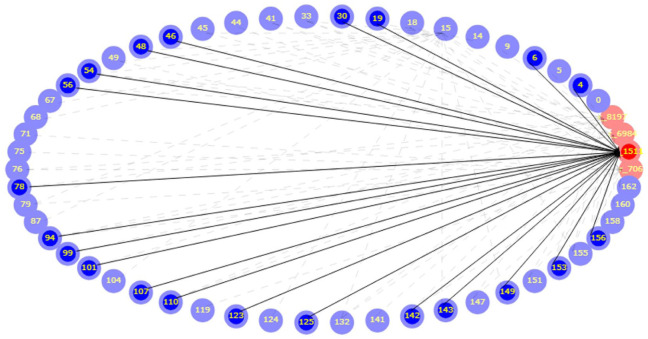
Citation analysis between documents (blue nodes) and citations (red nodes).

Figure 12 shows a citation network, with the help of Adjacency Analysis, centered on the most cited reference in the corpus (r_1511), which is authored by Chersich et al. (2020). Blue nodes are documents from the dataset and red nodes are the references they cite; arrows run from the citing document to the cited source. The hub-and-spoke pattern around (r_1511; Chersich et al., 2020) indicates that a large share of recent studies draws directly on this reference. The document “r_1511” (Chersich et al., 2020) is cited by “4” (Afzal et al., 2024), “6” (van Daalen et al., 2021), “19” (Spencer et al., 2022), “30” (Bouverat et al., 2024), “46” (Bonell et al., 2022), “48” (Scorgie et al., 2023), “54” (Conway et al., 2024), “56” (Zhao et al., 2023), “78” (Samuels et al., 2022), “94” (Khosravipour & Golbabaei, 2024), “99” (Doyle, 2023), “101” (Qiu et al., 2023), “107” (Tong et al., 2024), “110” (Yang et al., 2022), “123” (Wang et al., 2024), “125” (Nyadanu et al., 2022), “142” (Wu et al., 2023), “143” (Bansal et al., 2023), “149” (Part et al., 2022), “153” (Rancière et al., 2024), and “156” (de Bont et al., 2022). This confirms its foundational role for methods and concepts in women’s health and climate change. Examining the cluster of citing documents helps identify which subtopics rely on this source, how its arguments are extended or tested, and where subsequent work diverges. In practical terms, the figure flags (r_1511; Chersich et al., 2020) as a touchstone for the field and points to a coherent community of papers that build on it, which is useful for framing the literature review and for locating potential gaps that are not yet connected to this core.

**Figure 13.**
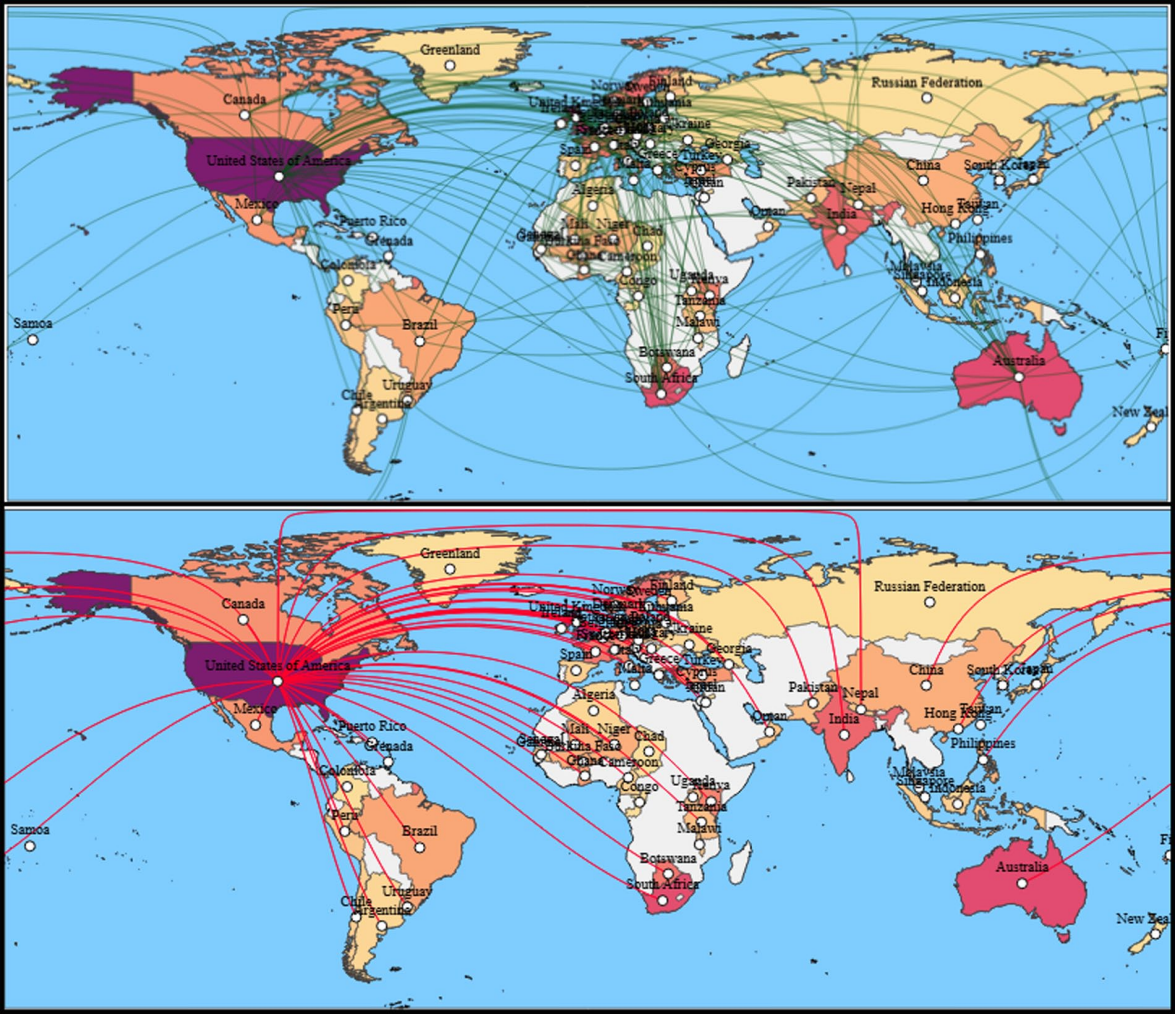
Collaboration analysis between countries and influence of the United States in the collaboration network.

Figure 13 (upper) shows the global collaboration network and how research is distributed across countries and linked through coauthorship ties. Collaborations are highly concentrated among institutions in the Global North, with the United States, the United Kingdom, and Australia emerge as central hubs that maintain dense partnerships with multiple regions. China and South Africa also appear as important nodes, which reflect their growing visibility in the field. By contrast, contributions from many climate-vulnerable countries in Africa, South Asia, and Latin America remain limited and are often connected to Northern partners rather than forming autonomous research clusters. The pattern is the sign of both the international scope of the field and the structural imbalances within it, where research leadership and agenda-setting are concentrated in well-resourced Northern institutions while Southern countries frequently provide the empirical contexts but remain underrepresented in authorship networks.

Figure 13 (bottom) shows that United States is the most dominant actor, connected to a wide range of countries across all regions. Its high publication volume and its capacity to shape research agendas through international partnerships is extensive. In other words, it is a structural imbalance: While the United States maintains links with many countries in the Global South, these relationships are often asymmetrical, with the United States occupying the central position of agenda-setter and resource provider, while Southern partners contribute primarily as sites of empirical data. It is a prime example that how research power is concentrated in well-resourced Northern institutions, reinforcing global hierarchies in knowledge production and underscoring the need for more equitable collaboration that recognizes and integrates local expertise.

**Figure 14.**
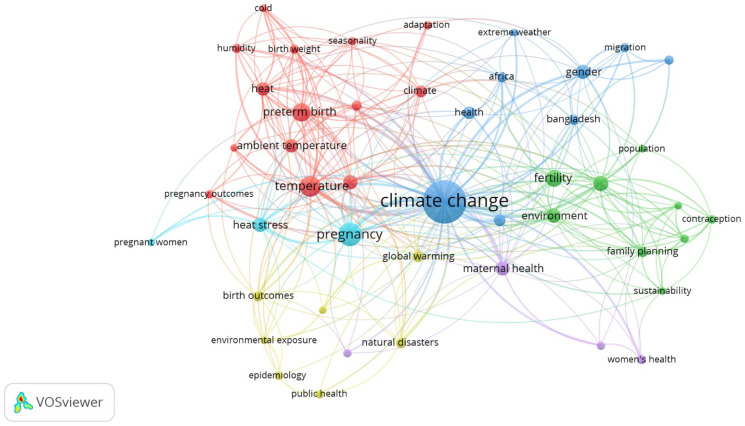
Network analysis of author keywords.

Figure 14 shows a keyword co-occurrence network based on author keywords, with a minimum threshold of five occurrences per keyword. Out of 960 author keywords, only 46 meet the criteria. The map shows six interconnected thematic clusters. The word “climate change” occurred 148 times, “temperature” 34 times, “pregnancy” 43 times, “preterm birth” 27 times, “fertility” 27 times, and “reproductive health” 18 times. The all-other words occurred 17 or less than 17 times. The first group is represented by the color red. Among the words in this group, “heat,” “preterm birth,” “ambient temperature,” and “temperature” are the most notable. The second group is represented by the color blue, where terms such as “climate change,” “gender,” “migration,” “Africa,” and “Bangladesh” appear frequently. This group highlights the role of social vulnerability and geographic contexts. The third group, shown in green, includes words such as “fertility,” “family planning,” “contraception,” and “sustainability,” “family planning” linking reproductive health concerns with broader demographic and development agendas. The fourth group, shown in purple, is organized around “maternal health,” “environmental health,” and “women’s health,” pointing to system-level approaches that situate women’s health within disaster risk and health system frameworks. The fifth group, represented in yellow, includes “epidemiology,” “environmental exposure,” and “birth outcomes,” which capture methodological orientations and outcome-specific studies. In the end, the sixth group shown in light blue (cyan) color included words like “heat stress,” “pregnancy,” and “pregnant women.”

**Figure 15.**
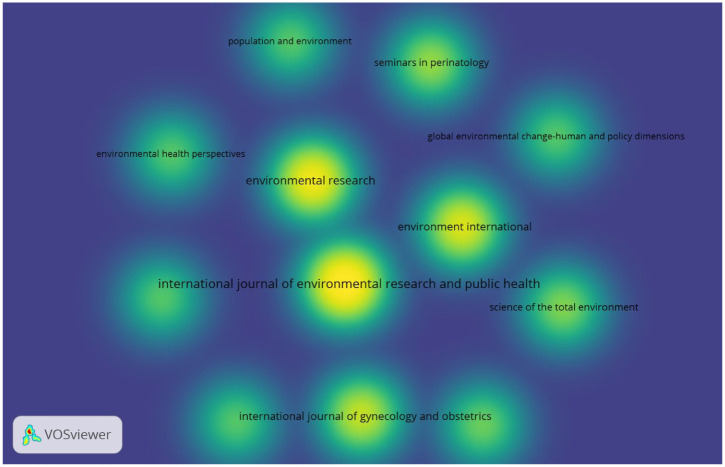
Bibliographic coupling of the journals (density visualization).

Bibliographic coupling of the journals is presented with density visualization in Figure 15. As inclusion criteria, a journal’s minimum number of publications was set to 5. Out of 207 sources, 12 journals met the threshold. For these 12 journals, the number of documents, citations, and the total link strength of bibliographic coupling with other journals were calculated. In Figure 8, each circle represents a journal, and the density was illustrated with color gradients. The shift from green to yellow indicates higher numbers of publications and greater density. The leading journal is the *International Journal of Environmental Research and Public Health* with 21 publications, 741 citations, and 1806 total link strength. It is followed by *Environmental Research* (16; 508; 1456), *Environment International* (13; 615; 1222), and the *International Journal of Gynecology and Obstetrics* (11; 140; 1056). Other journals include *Seminars in Perinatology* (8; 69; 792), *Science of the Total Environment* (7; 205; 700), *BJOG: An International Journal of Obstetrics & Gynaecology* (6; 169; 606), *Environmental Health Perspectives* (5; 297; 510), *Frontiers in Public Health* (5; 47; 510), *Global Environmental Change – Human and Policy Dimensions* (5; 335; 510), *PLoS One* (5; 106; 510), and *Population and Environment* (5; 49; 510). For all journals, the first number refers to publications, the second to citations, and the third to total link strength.

## Discussion

This bibliometric analysis provides a comprehensive overview of how research on climate change and women’s reproductive and maternal health has evolved, consolidated, and diversified over the past two decades. By tracing thematic trends, geographic distributions, author networks, and journal linkages, the findings highlight both the maturation of the field and its enduring asymmetries. On the one hand, the literature demonstrates increasing sophistication, with a clear shift from broad environmental exposures toward specific biomedical outcomes such as preterm birth, maternal health risks, and fertility. On the other hand, the analysis reveals structural imbalances, including the dominance of high-income countries in knowledge production, the central role of a few highly cited references in shaping intellectual trajectories, and the underrepresentation of justice-oriented, community-based, and Global South perspectives. Together, these findings underscore both the scientific progress and the epistemic gaps that define this rapidly expanding area of inquiry, pointing to opportunities for a more inclusive and interdisciplinary research agenda.

The analysis of abstracts (Figure 1) shows that research on climate change and women’s health is predominantly structured around a biomedical framing. Central terms such as *pregnancy, preterm birth, fertility, reproductive health*, and *maternal health* reflect the field’s emphasis on quantifiable health outcomes associated with environmental exposures. This pattern is consistent with the epidemiological literature documenting associations between heat, air pollution, and pregnancy complications.^7–9^ The dominance of *preterm birth* in trigram frequency underscores how certain outcomes have become sentinel indicators in this domain, aligning with meta-analyses that highlight its robustness as a climate-sensitive reproductive risk.^4^

Yet, the relative absence of terms related to *justice, inequality*, or *adaptation* indicates a thematic imbalance. While biomedical impacts dominate, socio-structural determinants are marginalized. This reflects broader critiques that global health research tends to privilege standardized, measurable endpoints over intersectional and feminist analyses of vulnerability.^14,42^ Bibliometrically, such clustering around biomedical outcomes may also mirror the influence of Northern institutions and funders that prioritize evidence legible to policy and clinical practice.^28^

The field is too focused on biomedical outcomes (like preterm birth) and is neglecting the crucial socio-structural determinants of health. This thematic imbalance means that measurable, clinical endpoints are prioritized over justice and inequality. For instance, studies from the Global South show that structurally vulnerable women (like wives whose husbands have migrated) have poor reproductive health and struggle to access care, revealing a significant gap in research that accounts for real-world constraints.^43,44^

### Temporal evolution of research keywords

The temporal evolution of keywords (Figures 2 and 3) demonstrates how the field has matured over the past decade. In 2020–2021, keywords focused on broad environmental descriptors (*humidity, temperature, global warming*), reflecting an exploratory phase. By 2022–2023, more specific biomedical terms (*pregnancy, reproductive health, air pollution*) appeared, marking a pivot toward outcome-oriented research. By 2024, the vocabulary further specialized with *fertility, maternal health*, and *preterm birth* at the center.

This progression is consistent with bibliometric trajectories observed in other emerging fields: Early agenda-setting emphasizes broad environmental contexts, while later phases crystallize around biomedical outcomes once epidemiological evidence strengthens.^29^ The rising prominence of reproductive endpoints corresponds with the growing epidemiological consensus linking climate exposures to adverse maternal and neonatal outcomes.^7,8^ However, justice-oriented and adaptation-related keywords remain underrepresented, underscoring the persistence of a biomedical focus and the limited uptake of feminist or decolonial frameworks.^14^

### Growth dynamics and field consolidation

The longitudinal keyword analysis (Figure 3) shows limited research activity prior to 2010, with three peaks in 2014, 2017, and 2020—periods that align with influential reviews and growing recognition of climate-sensitive reproductive health risks.^7,9^ The sharp growth after 2021 reflects both the COVID-19 pandemic’s role in amplifying awareness of health system vulnerabilities and the institutional mainstreaming of climate–health linkages. The persistence of *climate change* as an anchor keyword across all years reflects the field’s environmental health foundation, yet the limited presence of *gender* and *equity* suggests that broader social dimensions have not become mainstream.

### Global asymmetries in knowledge production

Figures 4 and 5 highlight persistent inequalities in the geography of knowledge production. Prominent Northern scholars such as Véronique Filippi, Ana Bonell, and Sari Kovats are highly connected to themes including *climate change, pregnancy, heat*, and *heat stress.* Their affiliations with institutions in the United States, United Kingdom, and Australia illustrate how intellectual authority is concentrated in high-income settings. Meanwhile, research from Zimbabwe, Pakistan, Bangladesh, Burkina Faso, and South Africa tends to focus on applied or context-specific topics such as *maternal health, hypertension*, and *occupational heat stress.* This distribution reproduces a colonial division of academic labor, whereby the Global North retains epistemic authority, while the Global South provides empirical case material.^18,45^

The country-level keyword distribution (Figure 6) further confirms this imbalance. The United States dominates, with high frequencies of *climate change, pregnancy, temperature, preterm birth*, and *air pollution.* China’s contributions emphasize *temperature, climate change*, and *air pollution*, reflecting its focus on environmental exposures. The United Kingdom emphasizes *pregnancy* and *heat stress*, while Australia highlights *pregnancy* alongside *climate change.* France contributes minimally, with dispersed keywords and no thematic consolidation. These patterns not only reflect regional research priorities shaped by funding landscapes and institutional infrastructures but also reveal underrepresentation of the Global South, despite its disproportionate vulnerability.^42^

### Author productivity and intellectual leadership

The productivity patterns of the top 20 authors (Figure 7) show a clear distinction between early pioneers and recent entrants. Foundational figures such as Sari Kovats and Rupa Basu were among the earliest to establish environmental epidemiology linkages, with work dating back to 2009–2010.^6,46^ Their sustained productivity highlights the centrality of epidemiology in shaping the field. However, most authors entered after 2016, with marked expansion from 2020 onward, corresponding to the mainstreaming of climate–health in policy discourse.^1^

From a bibliometric perspective, this surge reflects the field’s rapid maturation as younger scholars build upon the foundations laid by earlier epidemiologists. Yet, the dominance of Northern authors among the most productive reinforces epistemic asymmetries, with Southern scholars often excluded from leadership roles even when their regions serve as empirical sites of study.^14^

### Geographic distribution and global inequalities

The global productivity map (Figure 8) underscores the structural imbalance of knowledge production. Output is concentrated in high-income countries—particularly the United States, United Kingdom, China, and Australia—while much of Africa, South America, and parts of Asia remain underrepresented. This mirrors broader patterns in global health where epistemic authority is consolidated in the North, while the South is framed primarily as a site of risk.^18^

Such imbalances have profound implications. They risk producing one-size-fits-all recommendations derived from Northern epistemologies, which may fail to capture the lived experiences and adaptation strategies of women in climate-vulnerable regions. They also marginalize feminist, Indigenous, and community-based knowledge systems that could provide richer insights into resilience and justice-oriented adaptation.^25,42^ Addressing these asymmetries requires shifting funding flows, building South-led consortia, and embedding equity and justice in bibliometric and policy frameworks.

These geographic patterns are not coincidental but reflect structural epistemic injustice. The concentration of research output in high-income countries despite climate vulnerabilities being greatest in the Global South exemplifies what de Sousa Santos^18^ terms “epistemicide”—the systematic suppression and marginalization of knowledge produced in the South. This occurs through multiple mechanisms: Northern institutions control funding streams and thus research agendas; peer review and editorial processes are dominated by Northern scholars who may prioritize methodologies and frameworks familiar to them; English-language publication requirements disadvantage non-Anglophone scholars; and institutional resources (time, infrastructure, bibliometric tools) necessary for competitive scholarship are unequally distributed. From a FPE perspective, this epistemic inequality means that knowledge about women’s climate vulnerabilities is produced primarily by those who do not experience these vulnerabilities firsthand, risking abstraction from lived realities and the reproduction of Northern epistemological frameworks that may not capture Southern women’s agency, resilience, and knowledge systems

### Institutional and journal productivity

Figure 8 reveals the central role of a small cluster of institutions in sustaining research output. The London School of Hygiene and Tropical Medicine and the University of California are the most consistent contributors, underscoring the dominance of established Northern centers. However, recent growth from Peking University, Sun Yat-sen University, and Monash University suggests an expanding role for Chinese and Australian institutions in shaping the field. This diversification of research leadership indicates that while epistemic authority remains anchored in the North, new hubs are emerging that could shift the balance of knowledge production.

Similarly, Figure 9 shows that journal productivity is concentrated in a small set of outlets. The *International Journal of Environmental Research and Public Health* and *Environmental Research* are leading publication venues, with contributions also clustered in *Environmental International*, *BJOG: An International Journal of Obstetrics and Gynaecology*, and the *International Journal of Gynecology and Obstetrics.* More recent activity in *Frontiers in Public Health* and *Seminars in Perinatology* suggests a diversification of publication spaces, potentially driven by faster review processes and open access dynamics. This diversification not only expands accessibility but also raises questions about the uneven prestige hierarchies across journals and the influence of publication venues on shaping thematic focus.

### Intellectual canon and most cited references

The table of most cited references highlights the intellectual canon of the field. Chersich et al. (2020) stands as the most cited, consolidating the evidence that high temperatures increase risks of preterm birth, low birth weight, and stillbirths. Seminal works by Strand (2011) and Zhang et al. (2017) also emphasize temperature and seasonality, forming a consistent epidemiological base. Basu et al. (2010) and Strand et al. (2012) established early links between ambient heat and preterm delivery, while Bekkar et al. (2020) and He et al. (2016) expanded this evidence across geographies. Historical and broader analyses, such as Bruckner et al. (2014) and Deschenes et al. (2009), contextualize these risks within both long-term climate variability and socioeconomic dimensions. Collectively, this canon demonstrates how temperature and air pollution emerged as central pathways, shaping the biomedical dominance in the field’s thematic evolution.

### Collaboration and citation networks

The author collaboration networks (Figure 11) reveal that women scholars are at the forefront of research linking climate change and reproductive health. Central figures such as Kovats, Bonell, Roos, Nakstad, and Filippi anchor extensive collaborations, with Bonell’s network spanning particularly wide institutional and geographic boundaries. Kovats and Filippi are embedded in dense clusters with other leading figures such as Chersich and Hajat, suggesting their integration into long-standing epistemic communities. By contrast, Roos and Nakstad show more project-based collaborations, indicating valuable but less sustained integration into global consortia. Collectively, these networks underscore both the leadership of female scholars in shaping the field and the importance of collaboration density in amplifying research visibility and influence.

The citation analysis (Figure 12) demonstrates the field’s intellectual consolidation around Chersich et al. (2020), which functions as a touchstone for subsequent studies. The hub-and-spoke structure centered on this systematic review shows its foundational role in establishing methodological and conceptual baselines for linking high temperatures with adverse maternal and neonatal outcomes. The wide range of citing documents, from epidemiological syntheses^4^ to justice-oriented critiques,^42,45^ illustrates how this reference underpins both biomedical and socio-structural strands of scholarship. Importantly, the coherence of this cluster also highlights potential blind spots: Emerging themes such as adaptation, intersectionality, and Indigenous knowledge remain less connected to this canonical work. As such, Figure 12 not only confirms the centrality of Chersich et al. (2020) but also maps the boundaries of what has been canonized, offering guidance on where future research could diverge or innovate.

The global collaboration networks (Figure 13) demonstrate both the international reach and structural inequalities of the field. Research leadership is concentrated in the Global North, particularly the United States, the United Kingdom, and Australia, which dominate coauthorship ties and agenda-setting. China and South Africa also emerge as increasingly visible hubs, but much of Africa, South Asia, and Latin America remain underrepresented and often connected asymmetrically to Northern partners. The bottom map further highlights the centrality of the United States, whose extensive links underscore its role as both a resource provider and agenda-setter. While these collaborations expand the field’s global scope, they risk reproducing colonial hierarchies of knowledge production in which Southern partners function primarily as sites of data extraction rather than as equal co-creators of theory and policy.^14,18^

The keyword co-occurrence network (Figure 14) illustrates the thematic consolidation of the field into six clusters. The biomedical cluster (red) highlights the dominance of epidemiological concerns such as *heat, temperature, preterm birth*, and *ambient exposure.* The social vulnerability cluster (blue) links *climate change* to *gender, migration*, and regional identifiers such as *Africa* and *Bangladesh*, reflecting attention to inequities in exposure and resilience. The demographic-development cluster (green) connects *fertility, family planning*, and *sustainability*, highlighting how reproductive health intersects with broader demographic transitions. Purple nodes situate *maternal health* within systems-level framings of environmental and women’s health, while the yellow and cyan clusters reflect methodological orientations (*epidemiology, environmental exposure*) and outcome-specific studies (*pregnancy, heat stress*). This mapping reveals a maturing but still siloed field, where biomedical endpoints dominate, while structural and justice-oriented framings remain secondary.^47^

Finally, the bibliographic coupling of journals (Figure 15) underscores the centrality of a small set of outlets in shaping scholarly discourse. The *International Journal of Environmental Research and Public Health* emerges as the most influential hub, followed by *Environmental Research, Environment International*, and the *International Journal of Gynecology and Obstetrics.* Their high link strength indicates that they serve as intellectual crossroads for the field. Meanwhile, specialized outlets such as *Seminars in Perinatology* and *BJOG: An International Journal of Obstetrics and Gynaecology* provide disciplinary depth, and interdisciplinary journals such as *Global Environmental Change* introduce critical social science perspectives. The clustering of journals also highlights the dominance of environmental health and biomedical framings, suggesting that feminist, decolonial, and community psychology perspectives remain underrepresented in high-impact outlets. Diversifying the publication landscape to integrate such perspectives is critical for advancing equity and justice in climate–health research.

#### Structural mechanisms of epistemic inequality

The bibliometric patterns documented in this study are not accidental but reflect systematic structural mechanisms that concentrate knowledge production in the Global North. We identify five key mechanisms:

##### Funding architecture and research agenda-setting

Research funding for climate–health science is overwhelmingly concentrated in high-income countries. According to the Lancet Countdown,^1^ over 85% of climate–health research funding originates from institutions in North America, Europe, and Australia. This funding concentration has two effects: First, it determines which research questions are prioritized—Northern funders tend to support biomedical outcome research (preterm birth, fertility decline) that fits existing epidemiological frameworks, rather than structural or justice-oriented research that may challenge dominant paradigms. Second, funding requirements often mandate Northern institutional partnerships, positioning Southern institutions as data collection sites rather than as equal partners in conceptualization and analysis.^33^

The high output from the United States, United Kingdom, and Australia documented in Figures 8, 10, and 13 directly reflects this funding landscape. These countries host the major funding agencies (NIH, Wellcome Trust, NHMRC) that support climate–health research. Even when research is conducted in Southern contexts, the corresponding author—who typically controls publication and receives credit—is often affiliated with Northern institutions that hold the grants.

##### Publication infrastructure and language hegemony

The journals identified as most productive in our analysis (Figure 12)—*International Journal of Environmental Research and Public Health*, *Environmental Research*, *Environment International*—are all English-language, Northern-based outlets with editorial boards dominated by scholars from high-income countries. Publication in these journals requires: (a) fluency in academic English; (b) familiarity with Northern epistemological conventions and methodological standards; (c) resources for article processing charges (increasingly required in open-access models); and (d) time and institutional support for manuscript preparation.

These requirements systematically disadvantage Southern scholars. A recent analysis found that scholars from low- and middle-income countries face rejection rates 23% higher than those from high-income countries, even after controlling for study quality.^48^ Reviewers and editors, predominantly Northern-based, may undervalue methodologies (qualitative, participatory, community-based) more common in Southern research, or may question the “generalizability” of Southern case studies while accepting Northern studies as universal.

##### Institutional resources and infrastructure

The institutional productivity patterns (Figure 11) reveal concentration in well-resourced universities: London School of Hygiene and Tropical Medicine, University of California, Peking University. These institutions provide scholars with the following: protected research time (not dependent on clinical or teaching overload), access to bibliometric tools and databases, research support staff, computational infrastructure, and mentorship networks. Southern institutions, facing resource constraints, often cannot provide equivalent support, limiting scholars’ capacity to engage in time-intensive bibliometric or systematic review work.^49^

Moreover, the ability to access literature—essential for conducting bibliometric research itself—is unequally distributed. Institutions in high-income countries maintain subscriptions to expensive databases like WoS and Scopus, while many Southern universities cannot afford these, creating a knowledge access gap that reinforces publication disparities.

##### Citation cartels and epistemic authority

The citation patterns documented in Figure 13 and Table 3 reveal an intellectual hierarchy: Chersich et al. (2020), Strand (2011), and Zhang et al. (2017)—all Northern-led studies—function as canonical references. This is not necessarily because these studies are methodologically superior, but because: (a) they are published in high-impact journals with greater visibility; (b) Northern scholars are more likely to cite other Northern work due to network effects and familiarity; (c) systematic reviews led by Northern institutions carry perceived authority; and (d) citation advantage accumulates—highly cited work becomes more visible and thus more cited.

This creates a self-reinforcing cycle: Northern research gets cited more, which increases its perceived legitimacy, which leads to more citations. Southern scholarship, even when methodologically rigorous, may remain invisible if it is published in regional journals not indexed in major databases or written in languages other than English.^50^

##### Collaboration asymmetries and extractive partnerships

The collaboration networks (Figure 14) show extensive North–South connections, but network analysis alone cannot reveal power dynamics within these collaborations. Ethnographic research on global health partnerships reveals systematic asymmetries: Northern partners often control research design, data ownership, interpretation, and publication; Southern partners contribute data collection, local access, and implementation; authorship hierarchies frequently place Northern scholars in senior positions.^33^

The dominance of US-centered networks (Figure 15, bottom) exemplifies how collaboration can reproduce colonial dynamics. The United States maintains partnerships with numerous Southern countries, but these are often resource-dependent relationships where US institutions provide funding in exchange for data access. Southern scholars may be included as coauthors but lack control over theoretical framing, analytical decisions, or publication strategy. This represents what Bhakuni and Abimbola^16^ term “extractive internationalism”—the continuation of colonial-era extraction of resources, now extended to extraction of data and intellectual labor.

These structural mechanisms are mutually reinforcing and remarkably stable because they are embedded in institutions, funding structures, and disciplinary norms. Changing them requires not individual goodwill but systemic interventions: redistributing research funding to Southern institutions as principal investigators; decentering English and Northern journals in evaluation criteria; building Southern bibliometric infrastructure; and mandating equitable authorship and leadership in international collaborations. Without such interventions, the patterns documented in this study will persist.

### Policy recommendations for epistemic justice

Achieving epistemic justice in climate–health research requires coordinated structural reform across funding systems, publication practices, collaborations, and institutional evaluation. First, funders such as the NIH, Wellcome Trust, and the European Commission should redistribute resources by allocating at least 40% of climate–health funding to Southern institutions as principal investigators and revising eligibility criteria that currently marginalize them,^49^ while simultaneously investing in research infrastructure, bibliometric tools, and long-term institutional capacity. Funding priorities must also be decolonized by establishing Southern-led panels that center community-driven agendas focused on structural determinants, climate justice, and resilience. Second, publication systems should diversify editorial boards with a minimum of 40% Global South representation, expand multilingual publishing through translated abstracts and multilingual databases, and reform peer review standards to value qualitative, participatory, and Indigenous methodologies while addressing reviewer bias. Third, collaboration and authorship reforms are needed, including mandatory equitable authorship agreements guaranteeing Southern first or corresponding authorship, contributorship models that formally recognize data collection and community expertise, and ethical guidelines that prohibit extractive research practices lacking partnership or benefit-sharing. Fourth, academic institutions and funders must reform evaluation criteria to value regional and non-English publications and community-engaged work, build Southern capacity in bibliometrics and evidence synthesis, and strengthen South–South research networks such as those led by the African Academy of Sciences and Latin American climate justice consortia. Finally, accountability mechanisms should monitor epistemic equity through indicators such as grant leadership, authorship hierarchies, and citation patterns, with explicit equity targets and enforceable consequences, including funding restrictions for institutions that repeatedly perpetuate epistemic injustice.

### Limitations

WoS and Scopus were selected because they provide structured, high-quality bibliographic metadata needed for reliable computational analyses.^38^ Both databases apply established quality-control procedures and are widely used for citation analysis and network mapping. Combining WoS and Scopus is now a recommended practice to reduce disciplinary biases present in single-database approaches. In addition, both platforms offer robust APIs and export functions compatible with key bibliometric tools such as VOSviewer, Biblioshiny, and pyBibX. However, we acknowledge that this choice introduces significant selection bias with implications for the representation of Global South scholarship. PubMed, while focused on biomedical literature, indexes journals from low- and middle-income countries that may not be included in WoS or Scopus.^51^ Google Scholar, despite its methodological limitations for bibliometric analysis (lack of quality control, inconsistent metadata, difficulty in deduplication), indexes a far wider range of sources including regional journals, reports, and gray literature that often include Southern scholarship.^52^ Dimensions database, particularly through its integration with policy documents and datasets, captures research outputs beyond traditional journal articles.^53^

The exclusion of these databases likely results in underrepresentation of the following: (a) Southern journals not indexed in WoS/Scopus; (b) scholarship published in languages other than English; (c) interdisciplinary work published in regional outlets; and (d) community-based research that may appear in non-indexed venues. This is particularly problematic given our stated concern with epistemic justice, as our methodology may inadvertently reproduce the very exclusions we critique.

We proceeded with WoS and Scopus for three pragmatic reasons: First, our research questions centered on mapping the mainstream scientific discourse on climate change and women’s health as represented in internationally indexed literature, precisely to examine how this mainstream discourse is structured and whose voices dominate it. Second, including Google Scholar would have introduced insurmountable challenges in data quality, deduplication, and analysis due to its lack of standardization. Third, the computational bibliometric tools we employed (VOSviewer, Biblioshiny, pyBibX) are optimized for WoS/Scopus data formats.

## Conclusion

This bibliometric study provides a comprehensive mapping of global research on climate change and women’s reproductive and maternal health through an equity-focused lens, offering both a synthesis of thematic priorities and a critical reflection on the structural dynamics shaping the field. The findings reveal that scholarship has advanced significantly in recent years, consolidating around epidemiological evidence that links environmental stressors, particularly heat and air pollution, to adverse reproductive outcomes such as preterm birth, low birth weight, and maternal complications.

At the same time, the analysis underscores the uneven distribution of knowledge production. Research leadership is concentrated in high-income countries, particularly the United States, United Kingdom, China, and Australia, while contributions from climate-vulnerable regions in the Global South remain sparse and often embedded in asymmetrical North–South collaborations. Female scholars have played a central role in advancing the field, yet broader integration of intersectional, feminist, and community-driven perspectives is still limited. The dominance of a small cluster of journals and institutions further highlights epistemic concentration, raising concerns about the inclusivity of the research agenda.

Looking ahead, the challenge is not only to continue refining epidemiological evidence but also to expand the intellectual and geographic scope of the field. Future research must embed equity, justice, and local expertise at its core, ensuring that climate-vulnerable populations are not solely the subjects of study but also agents in shaping research agendas and adaptation strategies. Building South-led collaborations, diversifying publication venues, and integrating interdisciplinary perspectives, particularly from feminist, decolonial, and community psychology frameworks, are critical for advancing both the science and justice of climate–health research.

While the field has matured rapidly, its future progress will depend on overcoming entrenched imbalances in global knowledge production. Achieving this requires a conscious shift toward inclusive, equity-driven, and interdisciplinary approaches that align scientific inquiry with the lived realities of women most affected by the climate crisis.

## Supplemental Material

sj-docx-1-whe-10.1177_17455057261442096 – Supplemental material for Mapping two decades of research on climate change and women’s reproductive health: A bibliometric analysis (2000–2024) Supplemental material, sj-docx-1-whe-10.1177_17455057261442096 for Mapping two decades of research on climate change and women’s reproductive health: A bibliometric analysis (2000–2024) by Mudassar Aziz, Gulnaz Anjum and Abdul Rehman Nawaz in Women's Health

sj-docx-2-whe-10.1177_17455057261442096 – Supplemental material for Mapping two decades of research on climate change and women’s reproductive health: A bibliometric analysis (2000–2024) Supplemental material, sj-docx-2-whe-10.1177_17455057261442096 for Mapping two decades of research on climate change and women’s reproductive health: A bibliometric analysis (2000–2024) by Mudassar Aziz, Gulnaz Anjum and Abdul Rehman Nawaz in Women's Health
