# Supplementary material for: Mapping two decades of research on climate change and women’s reproductive health: A bibliometric analysis (2000–2024)
Source: Womens Health (Lond). 2026 Apr 17;22:17455057261442096. doi: 10.1177/17455057261442096 (PMC13100432; doi:10.1177/17455057261442096)

**Supplementary Material**

**Figure S1: Documents per Year**


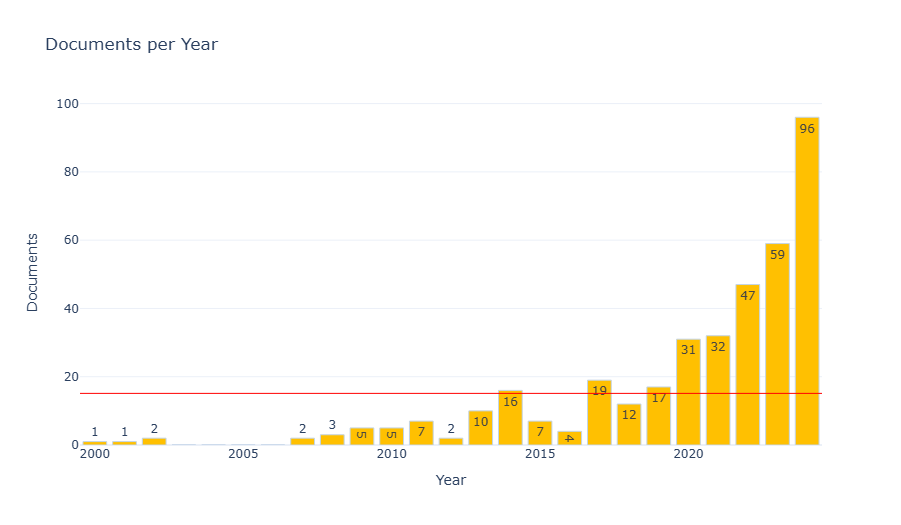


**Figure S2: Citation Per Year**


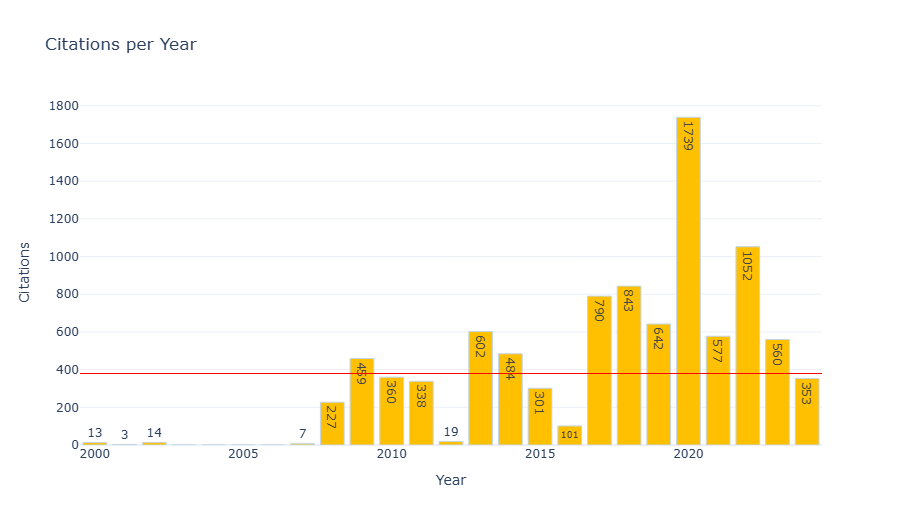


**Figure S3: Countries per citation**


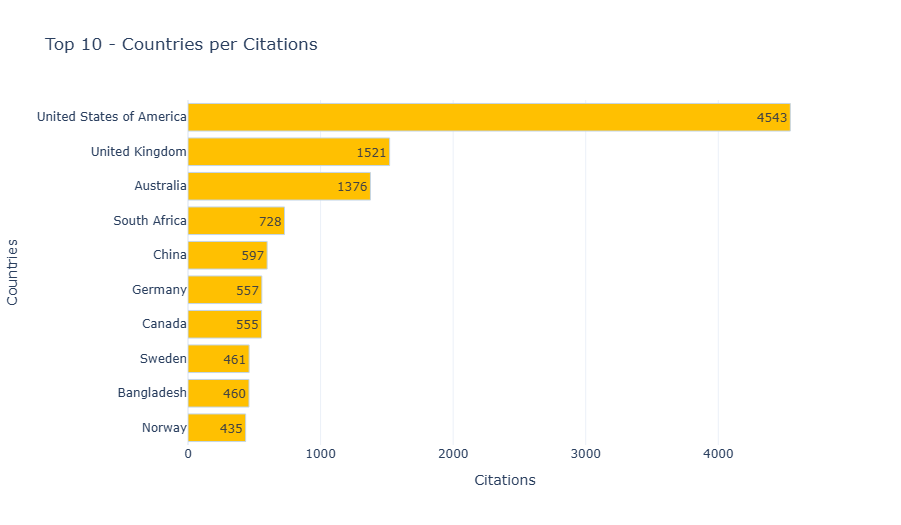
**Table S1: Word Cloud**

Based on the output of the previous cell, "Word 1" refers to the most important word found in the abstracts, and "Importance 1" is its corresponding importance score. The table is sorted in descending order of importance, so "Word 1" is the word with the highest calculated importance.

+---------------+--------------+---------------+--------------+-----------------+--------------+---------------+--------------+---------------+--------------+

| Word 1 | Importance 1 | Word 2 | Importance 2 | Word 3 | Importance 3 | Word 4 | Importance 4 | Word 5 | Importance 5 |

+---------------+--------------+---------------+--------------+-----------------+--------------+---------------+--------------+---------------+--------------+

| climate | 1.0 | health | 0.9714 | change | 0.8733 | women | 0.759 | temperature | 0.6994 |

| birth | 0.6398 | exposure | 0.5267 | pregnancy | 0.4658 | effect | 0.4534 | heat | 0.4472 |

| risk | 0.4273 | population | 0.3752 | impact | 0.3677 | study | 0.3503 | outcome | 0.3304 |

| environmental | 0.3205 | maternal | 0.2783 | reproductive | 0.2571 | preterm | 0.2484 | result | 0.2422 |

| association | 0.2385 | associated | 0.2335 | data | 0.2323 | pregnant | 0.2236 | increase | 0.2224 |

| extreme | 0.2211 | fertility | 0.1975 | studies | 0.195 | factor | 0.1938 | will | 0.1901 |

| increased | 0.1888 | high | 0.1888 | research | 0.1876 | level | 0.1826 | method | 0.1814 |

| children | 0.1776 | may | 0.1776 | low | 0.1739 | using | 0.1714 | review | 0.1689 |

| global | 0.1652 | air | 0.1627 | ci | 0.1602 | model | 0.1578 | adverse | 0.1553 |

| human | 0.1503 | related | 0.1503 | food | 0.1453 | analysis | 0.1453 | week | 0.1453 |

| among | 0.1441 | significant | 0.1416 | age | 0.1416 | term | 0.1404 | stress | 0.1366 |

| including | 0.1342 | based | 0.1329 | time | 0.1317 | ambient | 0.1304 | c | 0.1292 |

| development | 0.1267 | female | 0.1267 | well | 0.1255 | s | 0.1255 | gender | 0.1217 |

| stillbirth | 0.1217 | condition | 0.1193 | evidence | 0.118 | year | 0.1168 | day | 0.1168 |

| right | 0.1168 | pollution | 0.1155 | weather | 0.113 | countries | 0.113 | conducted | 0.113 |

| rate | 0.113 | period | 0.1118 | relationship | 0.1106 | higher | 0.1093 | used | 0.1093 |

| family | 0.1081 | vulnerable | 0.1068 | one | 0.1068 | child | 0.1056 | education | 0.1056 |

| region | 0.1056 | event | 0.1056 | finding | 0.1056 | disease | 0.1043 | weight | 0.1043 |

| ratio | 0.1043 | use | 0.1019 | individual | 0.1006 | background | 0.0994 | delivery | 0.0981 |

| infant | 0.0969 | season | 0.0957 | need | 0.0957 | affect | 0.0957 | number | 0.0944 |

| article | 0.0944 | included | 0.0944 | area | 0.0944 | planning | 0.0932 | due | 0.0932 |

| mother | 0.0932 | future | 0.0907 | community | 0.0907 | male | 0.0907 | potential | 0.0907 |

| disaster | 0.0894 | growth | 0.0894 | strategies | 0.0894 | month | 0.0894 | care | 0.0882 |

| social | 0.0882 | conclusion | 0.0882 | access | 0.0882 | people | 0.087 | environment | 0.0857 |

| gestational | 0.0857 | across | 0.0845 | early | 0.0845 | average | 0.0845 | cold | 0.0845 |

| author | 0.0845 | objective | 0.0832 | intervention | 0.082 | literature | 0.0807 | household | 0.0795 |

| fetal | 0.0795 | healthcare | 0.0795 | case | 0.0795 | migration | 0.0783 | two | 0.0783 |

| policy | 0.0783 | regression | 0.0783 | particularly | 0.077 | natural | 0.077 | policies | 0.077 |

| found | 0.077 | system | 0.077 | important | 0.0758 | increasing | 0.0758 | group | 0.0758 |

| challenge | 0.0758 | suggest | 0.0745 | sex | 0.0745 | participant | 0.0733 | trimester | 0.0733 |

| service | 0.0733 | identified | 0.072 | mortality | 0.072 | adaptation | 0.072 | paper | 0.0708 |

| different | 0.0708 | specific | 0.0708 | cause | 0.0708 | cohort | 0.0708 | water | 0.0696 |

| identify | 0.0696 | examine | 0.0696 | explore | 0.0683 | public | 0.0683 | observed | 0.0683 |

| issue | 0.0683 | heatwave | 0.0683 | within | 0.0671 | first | 0.0671 | limited | 0.0658 |

| resource | 0.0658 | reduce | 0.0646 | world | 0.0646 | influence | 0.0634 | role | 0.0634 |

| action | 0.0634 | total | 0.0634 | death | 0.0634 | analyse | 0.0634 | estimate | 0.0634 |

| provide | 0.0634 | daily | 0.0634 | ptb | 0.0634 | published | 0.0621 | international | 0.0621 |

| gestation | 0.0621 | non | 0.0621 | mean | 0.0621 | communities | 0.0609 | africa | 0.0609 |

| response | 0.0609 | security | 0.0596 | quality | 0.0596 | three | 0.0596 | survey | 0.0596 |

| mental | 0.0596 | affected | 0.0584 | economic | 0.0584 | measure | 0.0584 | support | 0.0584 |

| odds | 0.0584 | degree | 0.0584 | concern | 0.0584 | aim | 0.0584 | negative | 0.0571 |

| perinatal | 0.0571 | life | 0.0571 | seasonal | 0.0571 | practice | 0.0571 | relative | 0.0571 |

| long | 0.0571 | newborn | 0.0571 | birthweight | 0.0571 | experience | 0.0559 | estimated | 0.0559 |

| lower | 0.0559 | especially | 0.0547 | sexual | 0.0547 | current | 0.0547 | approach | 0.0547 |

| emission | 0.0547 | effort | 0.0534 | critical | 0.0534 | income | 0.0534 | significantly | 0.0534 |

| threat | 0.0534 | reported | 0.0534 | investigate | 0.0534 | less | 0.0522 | flood | 0.0522 |

| resilience | 0.0522 | per | 0.0522 | mechanism | 0.0522 | focus | 0.0509 | control | 0.0509 |

| linked | 0.0509 | pm2 | 0.0509 | wildfire | 0.0509 | disorder | 0.0509 | warming | 0.0497 |

| poor | 0.0497 | many | 0.0497 | compared | 0.0497 | existing | 0.0497 | interview | 0.0497 |

| g | 0.0497 | include | 0.0497 | likely | 0.0484 | range | 0.0484 | context | 0.0484 |

| key | 0.0484 | demographic | 0.0484 | exposed | 0.0484 | status | 0.0484 | reduction | 0.0484 |

| state | 0.0484 | lag | 0.0484 | pollutant | 0.0472 | consequence | 0.0472 | design | 0.0472 |

| overall | 0.046 | hot | 0.046 | four | 0.046 | size | 0.046 | humidity | 0.046 |

| major | 0.046 | examined | 0.046 | percentile | 0.046 | problem | 0.046 | variability | 0.0447 |

| new | 0.0447 | find | 0.0447 | carbon | 0.0447 | national | 0.0447 | mitigation | 0.0447 |

| face | 0.0447 | address | 0.0447 | qualitative | 0.0435 | decision | 0.0435 | show | 0.0435 |

| district | 0.0435 | complication | 0.0435 | local | 0.0422 | records | 0.0422 | discussion | 0.0422 |

| although | 0.0422 | highlight | 0.0422 | systematic | 0.0422 | understanding | 0.0422 | difference | 0.0422 |

| interval | 0.0422 | assess | 0.0422 | large | 0.041 | reserved | 0.041 | rural | 0.041 |

| greater | 0.041 | several | 0.041 | program | 0.041 | gap | 0.041 | cyclone | 0.041 |

| fire | 0.041 | severe | 0.0398 | agricultural | 0.0398 | variation | 0.0398 | information | 0.0398 |

| multiple | 0.0398 | elsevier | 0.0398 | cultural | 0.0398 | physical | 0.0398 | way | 0.0398 |

| confidence | 0.0398 | breastfeeding | 0.0398 | meteorological | 0.0398 | respectively | 0.0398 | u | 0.0398 |

| present | 0.0385 | work | 0.0385 | common | 0.0385 | meta | 0.0385 | incidence | 0.0385 |

| abortion | 0.0385 | adjusted | 0.0385 | whether | 0.0385 | implication | 0.0385 | prenatal | 0.0385 |

| source | 0.0385 | obstetric | 0.0385 | sample | 0.0373 | drought | 0.0373 | remain | 0.0373 |

| justice | 0.0373 | needed | 0.0373 | pre | 0.0373 | indigenous | 0.0373 | scale | 0.0373 |

| pattern | 0.0373 | knowledge | 0.036 | regarding | 0.036 | lead | 0.036 | sub | 0.036 |

| indicate | 0.036 | following | 0.036 | improve | 0.036 | rising | 0.036 | make | 0.036 |

| bangladesh | 0.036 | prevalence | 0.036 | database | 0.036 | e | 0.036 | linear | 0.036 |

| seasonality | 0.036 | girl | 0.036 | maximum | 0.036 | australia | 0.036 | n | 0.0348 |

| crisis | 0.0348 | available | 0.0348 | vulnerability | 0.0348 | spontaneous | 0.0348 | applied | 0.0348 |

| logistic | 0.0348 | farmers | 0.0348 | investigated | 0.0348 | performed | 0.0348 | midwives | 0.0348 |

| hazard | 0.0348 | smoke | 0.0348 | main | 0.0348 | patient | 0.0348 | lack | 0.0335 |

| young | 0.0335 | search | 0.0335 | childbearing | 0.0335 | men | 0.0335 | living | 0.0335 |

| south | 0.0335 | hypertension | 0.0335 | resulting | 0.0335 | pregnancies | 0.0335 | late | 0.0335 |

| utci | 0.0335 | link | 0.0335 | characteristics | 0.0335 | medical | 0.0335 | index | 0.0335 |

| setting | 0.0335 | benefit | 0.0335 | developing | 0.0323 | better | 0.0323 | aimed | 0.0323 |

| miscarriage | 0.0323 | science | 0.0323 | decline | 0.0323 | contribute | 0.0323 | born | 0.0323 |

| loss | 0.0323 | value | 0.0323 | labor | 0.0323 | relevant | 0.0323 | china | 0.0323 |

| singleton | 0.0323 | interaction | 0.0323 | lmic | 0.0323 | generation | 0.0311 | crucial | 0.0311 |

| production | 0.0311 | understand | 0.0311 | given | 0.0311 | effective | 0.0311 | socioeconomic | 0.0311 |

| part | 0.0311 | attention | 0.0311 | pressure | 0.0311 | reduced | 0.0311 | sensitive | 0.0311 |

| nutrition | 0.0311 | live | 0.0311 | matter | 0.0311 | premature | 0.0311 | window | 0.0311 |

| wave | 0.0311 | direct | 0.0298 | focused | 0.0298 | growing | 0.0298 | additional | 0.0298 |

| measured | 0.0298 | ltd | 0.0298 | elevated | 0.0298 | body | 0.0298 | might | 0.0298 |

| tool | 0.0298 | making | 0.0298 | century | 0.0298 | cost | 0.0298 | framework | 0.0298 |

| known | 0.0298 | particular | 0.0298 | reducing | 0.0298 | even | 0.0298 | hospital | 0.0298 |

| leading | 0.0298 | forest | 0.0298 | january | 0.0298 | distributed | 0.0298 | showed | 0.0298 |

| trend | 0.0298 | decade | 0.0298 | center | 0.0298 | stressor | 0.0298 | morbidity | 0.0286 |

| considered | 0.0286 | assessed | 0.0286 | saharan | 0.0286 | comprehensive | 0.0286 | general | 0.0286 |

| short | 0.0286 | must | 0.0286 | reproduction | 0.0286 | dynamic | 0.0286 | million | 0.0286 |

| insecurity | 0.0286 | decrease | 0.0286 | evaluate | 0.0286 | function | 0.0286 | later | 0.0286 |

| postnatal | 0.0286 | respiratory | 0.0286 | score | 0.0286 | past | 0.0273 | woman | 0.0273 |

| previous | 0.0273 | duration | 0.0273 | physiological | 0.0273 | collected | 0.0273 | programme | 0.0273 |

| united | 0.0273 | increasingly | 0.0273 | despite | 0.0273 | defined | 0.0273 | without | 0.0273 |

| childhood | 0.0273 | offspring | 0.0273 | assessment | 0.0273 | third | 0.0273 | society | 0.0273 |

| eclampsia | 0.0273 | organization | 0.0273 | last | 0.0273 | clinical | 0.0273 | sustainable | 0.0273 |

| indicator | 0.0273 | placental | 0.0273 | chronic | 0.0273 | pathway | 0.0273 | caused | 0.0261 |

| gendered | 0.0261 | pubmed | 0.0261 | consider | 0.0261 | greenhouse | 0.0261 | addressing | 0.0261 |

+---------------+--------------+---------------+--------------+-----------------+--------------+---------------+--------------+---------------+--------------+

**Figure S4: Tree Map of Author Keywords**


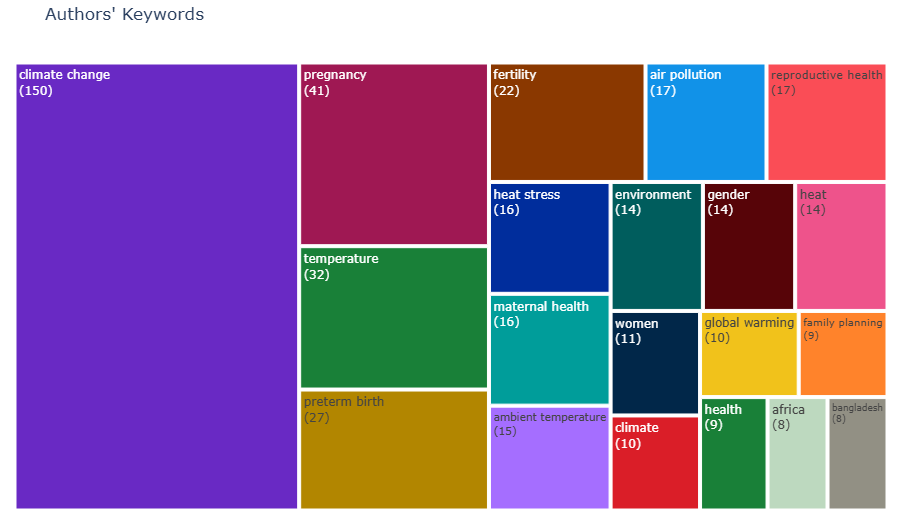


**Table S2: Trigram Analysis**

| **index** | **Word** | **Freq** |
| --- | --- | --- |
| 0 | risk preterm birth | 34 |
| 1 | effects climate change | 33 |
| 2 | impacts climate change | 29 |
| 3 | adverse pregnancy outcomes | 28 |
| 4 | adverse birth outcomes | 28 |

To identify commonly discussed themes in the literature, a 3-gram analysis was conducted on the abstracts of all retrieved articles. Prior to the n-gram extraction, the text corpus was normalized by removing standard English stopwords (e.g., *“the,” “and,” “to”*) as well as custom domain-specific words that frequently appeared across documents but did not contribute to thematic meaning.

rmv_custom_words = [

    # Generic stop/filler words

    "the", "and", "of", "to", "in", "on", "with", "by", "as", "for", "from",

    "at", "an", "this", "that", "these", "those", "it", "its", "they", "their",

    "them", "you", "your", "we", "our", "us", "has", "have", "was", "were", "been",

    "is", "are", "be", "being", "can", "could", "may", "might", "will", "would",

    "should", "than", "also", "not", "no", "yes", "do", "does", "did",

    # Academic boilerplate

    "study", "studies", "results", "conclusion", "suggest", "investigate",

    "conducted", "found", "observed", "reported", "indicate", "includes",

    "including", "data", "analysis", "based", "associated", "association",

    # Publishing noise

    "elsevier", "reserved", "rights", "available", "doi", "preprint"

]

**Figure S5: Overlay Visualization of author keywords**


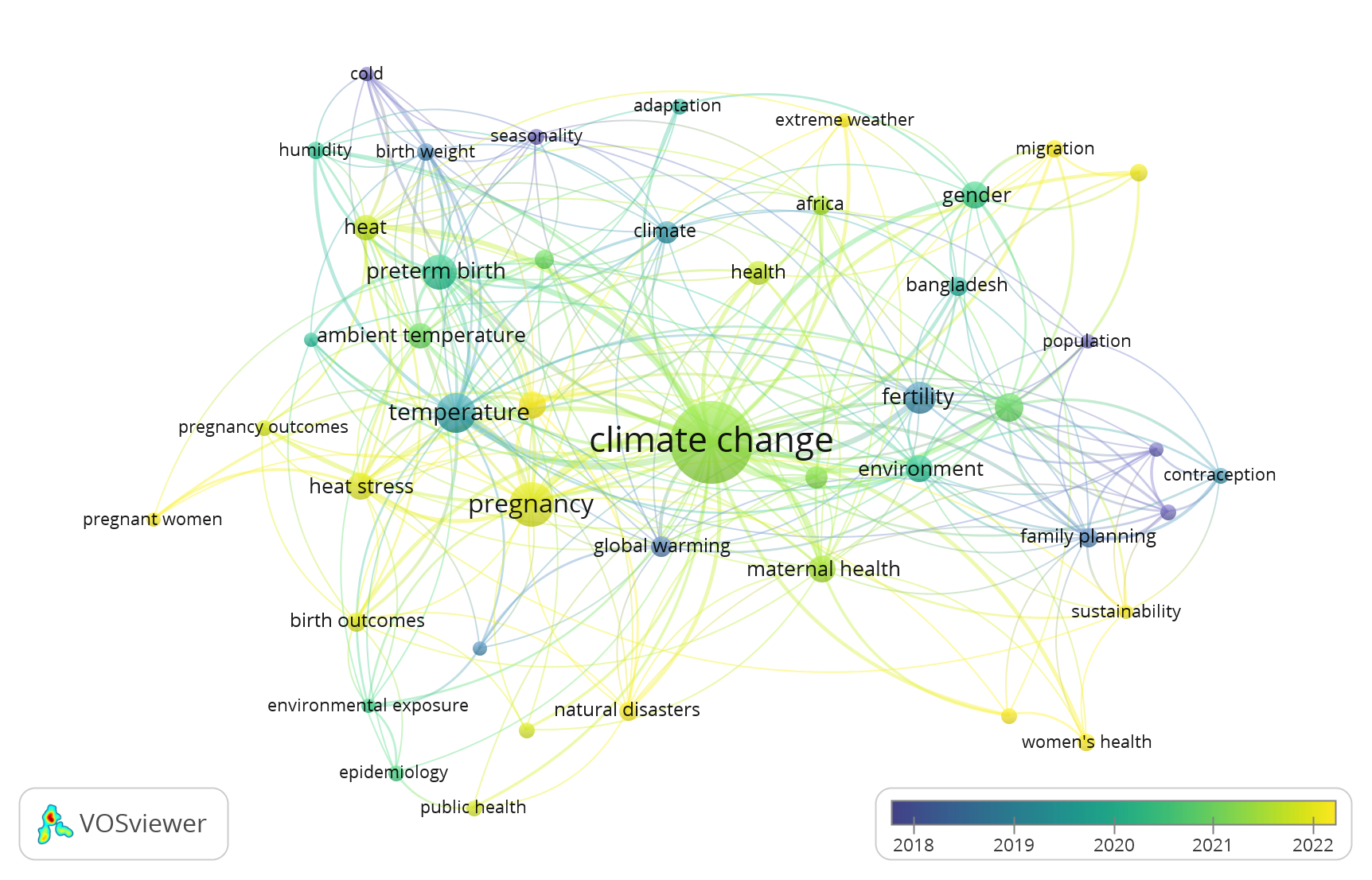


**Figure S6: Countries’ collaboration networks**


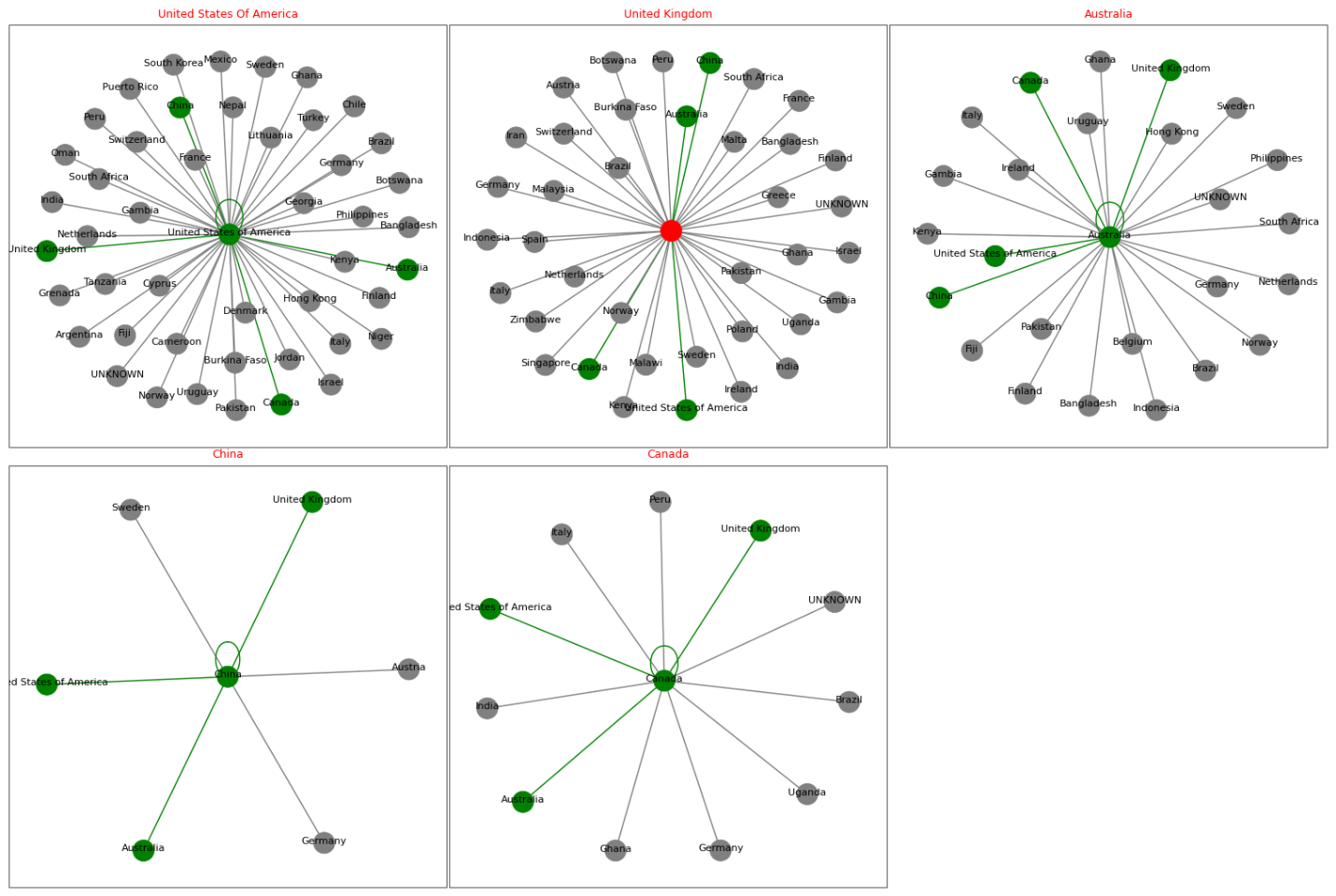

Supplement: sj-docx-2-whe-10.1177_17455057261442096 – Supplemental material for Mapping two decades of research on climate change and women’s reproductive health: A bibliometric analysis (2000–2024) [file sj-docx-2-whe-10.1177_17455057261442096.docx]
